# Genetic architecture of the white matter connectome of the human brain

**DOI:** 10.1126/sciadv.add2870

**Published:** 2023-02-17

**Authors:** Zhiqiang Sha, Dick Schijven, Simon E. Fisher, Clyde Francks

**Affiliations:** ^1^ Language and Genetics Department, Max Planck Institute for Psycholinguistics, Nijmegen, Netherlands.; ^2^ Donders Institute for Brain, Cognition and Behaviour, Radboud University, Nijmegen, Netherlands.; ^3^ Department of Human Genetics, Radboud University Medical Center, Nijmegen, Netherlands.

## Abstract

White matter tracts form the structural basis of large-scale brain networks. We applied brain-wide tractography to diffusion images from 30,810 adults (U.K. Biobank) and found significant heritability for 90 node-level and 851 edge-level network connectivity measures. Multivariate genome-wide association analyses identified 325 genetic loci, of which 80% had not been previously associated with brain metrics. Enrichment analyses implicated neurodevelopmental processes including neurogenesis, neural differentiation, neural migration, neural projection guidance, and axon development, as well as prenatal brain expression especially in stem cells, astrocytes, microglia, and neurons. The multivariate association profiles implicated 31 loci in connectivity between core regions of the left-hemisphere language network. Polygenic scores for psychiatric, neurological, and behavioral traits also showed significant multivariate associations with structural connectivity, each implicating distinct sets of brain regions with trait-relevant functional profiles. This large-scale mapping study revealed common genetic contributions to variation in the structural connectome of the human brain.

## Teaser

Variability in white matter connections of the human brain is associated with hundreds of newly discovered genomic loci.

## INTRODUCTION

Cognitive functions and behaviors are supported by dynamic interactions of neural signals within large-scale brain networks (*1*). Neural signals propagate along white matter connections that link cortical, subcortical, and cerebellar regions to form the structural connectome (*2*). White matter connections also modulate neural signals and distribute trophic factors between brain regions (*3*), helping to establish and maintain functional specialization of subnetworks. Various heritable psychiatric and neurological disorders can involve altered white matter structural connectivity, relating, for example, to cognitive deficits, clinical presentation, or recovery (*4*, *5*). It is therefore of great interest to understand which DNA variants, genes, and pathways affect white matter connections in the human brain, as they are likely to influence cognitive and behavioral variability in the population, as well as predisposition to brain disorders.

Diffusion tensor imaging (DTI) enables in vivo noninvasive study of white matter in the brain (*6*). This technique characterizes the diffusion of water molecules, which occurs preferentially in parallel to nerve fibers due to constraints imposed by axonal membranes and myelin sheaths (*7*). Metrics commonly derived from DTI, such as fractional anisotropy or mean diffusivity, reflect white matter microstructure and can index its integrity (*7*, *8*). In contrast, tractography involves defining white matter connections at the macroanatomical scale, which permits the measurement of connectivity strengths by counting the streamlines that link each pair of regions. Streamlines are constructed to pass through multiple adjacent voxels in DTI data, when the principal diffusion tensor per voxel aligns well with some of its direct neighbors (*9*). Tractography therefore produces subject-specific measures of regional interconnectivity that are ideally suited for brain network-level analysis.

Recently, genome-wide association studies (GWAS) have reported that a substantial proportion of interindividual variability in white matter microstructural measures can be explained by common genetic variants, with single-nucleotide polymorphism (SNP)–based heritabilities ranging from 22 to 66% (*10*, *11*). These studies also identified specific genomic loci associated with white matter microstructural measures (*10*, *11*). However, microstructural measures do not necessarily capture topological properties of macroscale brain networks, such as the total amount of structural connectivity between distant pairs of brain regions. In principal, interindividual variability in topological features of the white matter connectome may be influenced by genetic variants that are partly distinct from those that influence white matter microstructure. For example, genetic influences on axon outgrowth and guidance during the development of long-distance connections may be most detectable in terms of connection strengths as measured through tractography, without necessarily affecting the microstructural integrity of those connections. However, to our knowledge, nerve fiber tractography has not previously been used for large-scale genome-wide association analysis of brain structural networks, likely because of heavy computational requirements for running tractography in tens of thousands of individuals.

Here, we aimed to characterize the genetic architecture of white matter structural network connectivity in the human brain, using fiber tractography. DTI data from 30,810 participants of the U.K. Biobank adult population dataset were used to construct the brain-wide structural connectivity network of each individual. In combination with genome-wide genotype data, we then carried out a set of genetic analyses of tractography-derived metrics, in terms of the sum of white matter connectivity linking to each of 90 brain regions as network nodes and 947 connectivity measures as network edges linking specific pairs of regions. The total connectivity of a node (brain region) likely relates to its global role in information transfer within multiple subnetworks, whereas individual connections between specific pairs of regions are more locally restricted measures. We anticipated that genetic influences on node-level and edge-level network measures might therefore be partly distinct, where some genetic effects are more relevant at larger scales whereas others could affect relatively specific circuit components.

Our genetic analyses included SNP-based heritability estimation, multivariate GWAS (mvGWAS), and biological annotation of associated loci. Then, to illustrate how multivariate gene-brain associations arose in the data and how the brain-wide mvGWAS results could be queried in relation to any specific brain network of interest, we used the results to identify genomic loci that are associated with structural connections between core language–related regions of the left hemisphere. Various aspects of language function—especially related to language production—show strong hemispheric lateralization, with roughly 85% of people having left-hemisphere dominance (*12*).

Last, we assessed how genetic disposition to brain disorders and other behavioral traits manifests in terms of white matter connectivity in the general population. To do so, we mapped multivariate associations of the brain-wide, white matter tractography metrics with polygenic scores for a variety of heritable brain disorders or behavioral traits: schizophrenia, bipolar disorder, autism, attention-deficit hyperactivity disorder, left-handedness, Alzheimer’s disease, amyotrophic lateral sclerosis, and epilepsy. We annotated the resulting brain maps with cognitive functions, using large-scale meta-analyzed functional neuroimaging data, to describe aspects of brain function that may be affected by polygenic dispositions to different forms of neurodivergence in the general population.

## RESULTS

### White matter connectomes of 30,810 adults

**Fig. 1.**
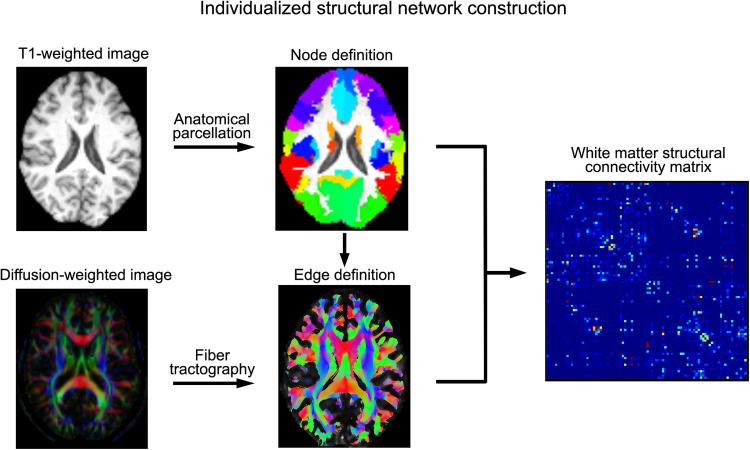
Schematic of white matter network construction within an individual brain. Network nodes were defined by mapping the Automated Anatomical Labeling atlas from common MNI space to individual space, with 45 regions per hemisphere (including cortical and subcortical structures). The edge between each pair of regions was defined as the number of streamlines constructed by tractography based on the corresponding diffusion tensor image, adjusted for the combined volume of the two connected regions. The process yielded a zero-diagonal symmetrical 90 × 90 undirected connectivity matrix for each of 30,810 participants (the top triangles were then used for subsequent analyses).

For each of 30,810 adult participants with diffusion magnetic resonance imaging (MRI) and genetic data after quality control, we performed deterministic fiber tractography (*9*) between each pair of regions defined in the Automated Anatomical Labeling atlas (*13*) (45 regions per hemisphere comprising cerebral cortical and subcortical structures) (Fig. 1 and Materials and Methods). In the structural connectivity matrix of each individual, each region was considered a node, and each connection between a pair of regions was considered an edge. We excluded edges when more than 20% of individuals had no streamlines connecting a given pair of regions, resulting in 947 network edges. To quantify a given edge in each individual, the streamline count for that edge was divided by the individual-specific gray matter volume of the two regions being connected (as larger regions tend to have more streamlines connecting to them). These volume-adjusted network edge measures were also used to calculate the node-level connectivity of each region, i.e., the sum of all volume-weighted edge measures connecting with a given region, for each participant. The resulting node and edge measures were adjusted for demographic and technical covariates and normalized across individuals (see the “Network construction and analysis” section), before being used for genetic analyses.

Of the 947 network edges, 377 connected pairs of left-hemisphere regions, 355 connected pairs of right-hemisphere regions, and 215 involved interhemispheric connections. The top 10% of regions in terms of connectivity included the supplementary motor cortex, precuneus, medial superior frontal cortex, and subcortical regions bilaterally—caudate and thalamus (fig. S1 and table S1). The latter observation is consistent with previous studies showing that subcortical regions connect widely with the cerebral cortex, to generate reciprocal cortical-subcortical interactions that together support many cognitive functions (*14*).

### Heritabilities of connectivity measures

**Fig. 2.**
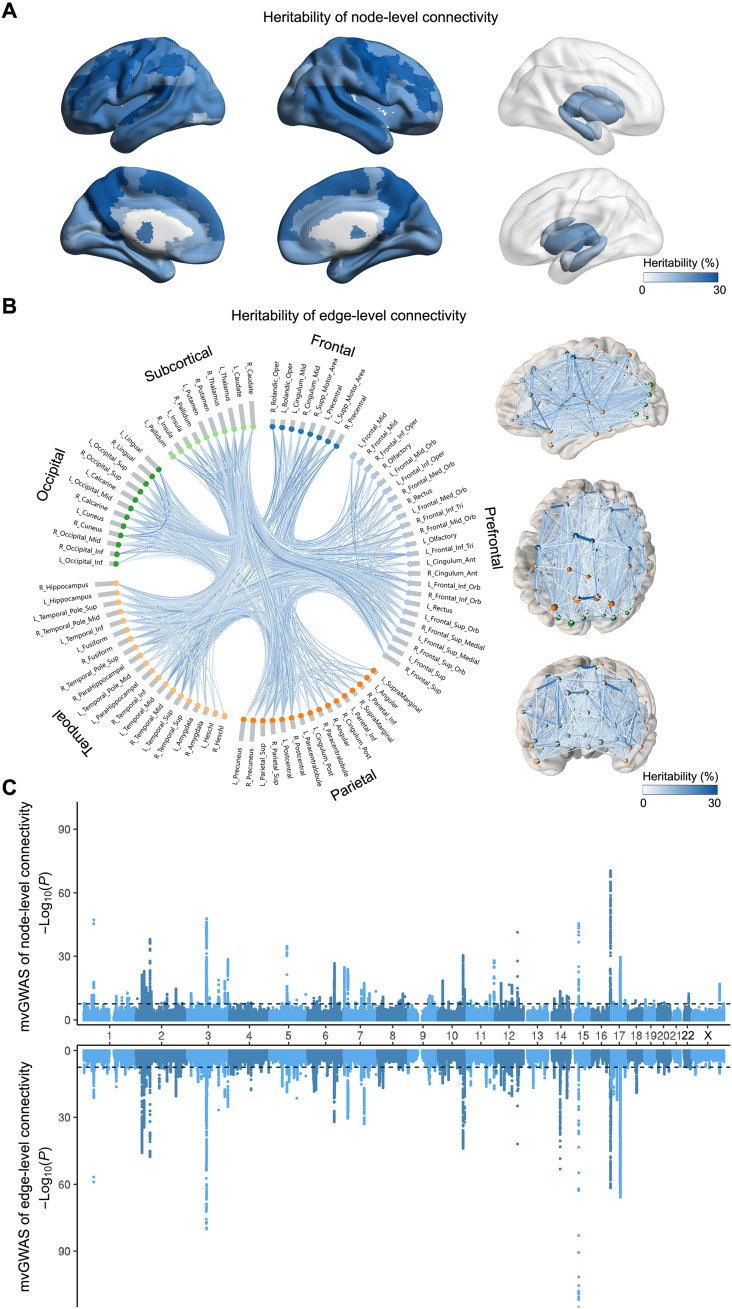
SNP-based heritability and mvGWAS analyses of node-level connectivity and edge-level connectivity in 30,810 participants. (**A**) All 90 node-level (i.e., regional) connectivities showed significant SNP-based heritability after Bonferroni correction, ranging from 7.8 to 29.5%. (**B**) Eight hundred fifty-one of 947 edge-level connectivities showed significant SNP-based heritability after Bonferroni correction, ranging from 4.6 to 29.5%. Right: Brain maps. Left: Nodes grouped by frontal, prefrontal, parietal, temporal, and occipital cortical lobes and subcortical structures. Heritabilities can be visualized interactively in a dynamic Web-based interface (see “Data and materials availability” statement). (**C**) Miami plot for mvGWAS of 90 node-level connectivities (top) and 851 edge-level connectivities (bottom). The black lines indicate the genome-wide significance threshold *P* < 2.5 × 10^−8^ (Materials and Methods).

The GCTA (Genome-wide Complex Trait Analysis) software (*15*) was used to estimate the SNP-based heritability (*h*^2^) for each network measure, that is, the extent to which variance in each connectivity measure was explained by common genetic variants across the autosomes (Materials and Methods). All of the 90 node-level (region-based) connectivity measures were significantly heritable (Bonferroni-corrected *P* < 0.05 after testing 90 measures), ranging from 7.8 to 29.5% (mean *h*^2^ = 18.5%; Fig. 2A, fig. S2, and table S2). Most homologous nodes of the left and right hemispheres showed similar heritabilities, but some nodes showed prominent differences of heritability between hemispheres, such as the inferior parietal cortex (left: 27.0% versus right: 19.42%), pars triangularis (left: 23.4% versus right: 16.9%), and inferior occipital cortex (left: 8.0% versus right: 15.7%; Fig. 2A and table S2). Eleven node-level connectivities showed *h*^2^ estimates of >25% (table S2), with the superior temporal cortex in the left hemisphere being the highest (*h*^2^ = 29.5%, *P* < 1 × 10^−20^).

Eight hundred fifty-one of 947 edge-level connectivities (i.e., connections between specific pairs of regions) showed significant heritability (Bonferroni-corrected *P* < 0.05 after testing 947 measures; fig. S2), ranging from 4.6 to 29.5% (mean, 9.6%). Eleven edges had *h*^2^ > 20%, primarily for connections linking frontal regions (e.g., superior and middle frontal cortex) and supplementary motor and occipital cortex (e.g., cuneus and lingual). The mean *h*^2^ was 9.9% for 351 edges within the left hemisphere, 10.0% for 333 edges within the right hemisphere, and 8.1% for 167 interhemispheric edges (Fig. 2B and tables S3 to S6). Across the 851 significantly heritable edges, heritability was not correlated with mean fiber length (rho = 0.01; fig. S3), suggesting that short-range and long-range white matter connections are similarly affected by genetic variation overall.

Reliability of heritable network measures was assessed using data from 1005 of the 30,810 individuals who had undergone brain scans on two separate occasions (Materials and Methods): Intraclass correlation coefficients (ICCs) based on the same processing pipeline applied to data from the first and second scanning visits had a median of 0.83 (range, 0.45 to 0.93) for the 90 heritable node-level measures and a median of 0.66 (range, 0.28 to 0.94) for the 851 heritable edge-level measures (fig. S4 and tables S7 and S8). Measurement reliability was positively correlated with heritability: *r* = 0.67 across 90 node-level connectivities and *r* = 0.60 across 851 heritable edge-level connectivities (fig. S4). This is consistent with the fact that measurement error is assigned to the “environmental” (i.e., nongenetic) component of trait variance in heritability analysis, so that less reliable measures tend to be less heritable. Regardless, we reasoned that all significantly heritable network measures had enough reliably measured variation to contribute to subsequent mvGWAS analysis. This is because detecting heritability relies on trait similarity being increased in pairs of individuals with higher genetic similarity and therefore necessitates at least a small proportion of trait variance being reliably measured across and within individuals, while single genetic loci are only expected to explain very small amounts of that heritable variance. The heritability data can be visualized interactively in a dynamic Web-based interface (see “Data and materials availability” statement).

### Multivariate genome-wide association analyses of white matter connectivity

The multivariate omnibus statistical test (MOSTest) software (*16*) was used to perform two separate mvGWAS analyses, first for the 90 node-level connectivity measures in a single multivariate genome-wide screen and then for the 851 edge-level connectivities in another single multivariate genome-wide screen, both times in relation to 9,803,735 SNPs spanning the genome. This analysis examined each SNP separately for its associations with multiple structural network measures, by simultaneously modeling the distributed nature of genetic influences across the brain (Materials and Methods). FUMA (*17*) was used to clump mvGWAS results on the basis of linkage disequilibrium (LD) and to identify independent lead SNPs at each associated genomic locus (Materials and Methods). At the *P* = 2.5 × 10^−8^ significance level (i.e., the standard GWAS significance threshold of *P* = 5 × 10^−8^ but Bonferroni-corrected for two mvGWAS), we identified 140 lead SNPs in 117 distinct genomic loci associated with node-level connectivities (Fig. 2C, fig. S5, and table S9) and 211 lead SNPs in 166 distinct genomic loci associated with edge-level connectivities (Fig. 2C, fig. S5, and table S10). Permutation analysis under the null hypothesis of no association indicated that MOSTest correctly controlled type I error (Materials and Methods and figs. S6 and S7). Except for chromosome 21, each chromosome had at least one locus associated with either node-level or edge-level connectivity.

Twenty-six lead SNPs were found in common between the node-level mvGWAS and edge-level mvGWAS. While a degree of overlap was to be expected given that the node-level metrics were computed from the edge-level metrics (i.e., are not independent), the fact that the large majority of lead SNPs were detected for either node-level or edge-level connectivity, but not both, supports the importance of performing genetic association analyses at these different network levels.

For each lead SNP, MOSTest indicated the contribution of each brain metric to its multivariate association, by reporting a *z* score derived from each metric’s univariate association with that SNP (Materials and Methods and tables S11 and S12). In the node-level mvGWAS, regions with the greatest magnitude *z* scores considered across all lead SNPs were the bilateral putamen (left mean |*z*| = 2.05, right mean |*z*| = 1.86), left pallidum (mean |*z*| = 2.02), bilateral middle frontal cortex (left mean |*z*| = 1.98, right mean |*z*| = 1.89), and middle cingulate cortex (mean |*z*| = 1.82; fig. S8 and table S13). For example, the left putamen, which had the highest overall contribution across lead SNPs (mean |*z*| = 2.05), was especially strongly associated with rs12146713 on 12q23.3 (*z* = −10.48), rs72748148 on 9q31.3 (*z* = 7.35), rs798528 on 7p22.3 (*z* = −6.74), rs7935166 on 11p11.2 (*z* = 6.22), and rs3795503 on 1q25.3 (*z* = 6.01; table S13).

In the mvGWAS of edge-level connectivity, edges that showed high magnitude *z* scores considered across all lead SNPs mainly connected the precuneus, calcarine, middle temporal, and pre- and postcentral cortex (table S14 and fig. S9). The edge linking the left and right precuneus had the greatest contribution across lead SNPs (mean |*z*| = 1.60) and was especially associated with the variants rs946711 on 10p12.31 (*z* = −5.58) and 3:190646282_TA_T on 3q28 (*z* = −5.53).

### The majority of genomic loci associated with structural connectivity were previously unidentified

Together, our node-level connectivity mvGWAS and edge-level connectivity mvGWAS identified 325 lead SNPs, of which only 101 were previously associated with at least one trait in the NHGRI-EBI GWAS catalog (tables S9 and S10) (*18*). This indicates that the majority (68.92%) of loci implicated here in the structural connectome were not identified by previous studies. There were 65 SNPs in common with those reported in previous GWAS of brain measures (*11*, *16*, *19*–*21*). Specifically, 46 of our lead SNPs were previously associated with brain regional volumes (*19*, *22*), 29 with regional cortical thicknesses (*16*, *21*), 33 with regional cortical surface areas (*21*, *23*), and 20 with white matter microstructure (*11*, *24*). Apart from brain measures, 11 of our lead SNPs were associated with mental health traits (e.g., autism, schizophrenia, and anxiety) (*25*, *26*), 11 of our lead SNPs with cognitive functions (e.g., cognitive ability and performance) (*27*, *28*), 4 of our lead SNPs with neurological diseases (e.g., Alzheimer’s disease and epilepsy) (*29*, *30*), and 40 of our lead SNPs with nonbrain physiological and physical variables (e.g., waist-hip ratio, cholesterol levels, and lung function) (*31*, *32*). In addition, we compared our results with those reported in a recent GWAS of white matter microstructure integrity for which the results have not been deposited in the GWAS catalog (*10*): Thirty-two of their lead SNPs overlapped with those from our mvGWAS analyses (table S15).

### Functional annotations of genomic loci associated with the structural connectome

We used FUMA (*17*) to annotate SNPs to genes at significantly associated loci by three strategies: physical position, expression quantitative trait locus (eQTL) information, and chromatin interactions. For the node-level connectivity mvGWAS, 879 unique genes were identified through these three strategies (table S16 and fig. S10). Ninety-five of 140 lead SNPs had at least one eQTL or chromatin interaction annotation, indicating that these variants (or other variants in high LD with them) affect gene expression. For example, rs7935166 on 11p11.2 (multivariate *z* = 5.71, *P* = 1.15 × 10^−8^) is intronic to *CD82*, which has been reported to promote oligodendrocyte differentiation and myelination of white matter (*33*). This lead SNP is a brain eQTL (*34*, *35*) of *CD82* and also shows evidence for cross-locus chromatin interaction via the promoter of *CD82* in adult brain (*34*). As another example, rs35396874 on 6q21 (multivariate *z* = 6.64, *P* = 3.17 × 10^−11^) affects the expression of its surrounding gene *FOXO3*, a core element of the TLR/AKT/FoxO3 pathway that is important for repairing white matter injury mediated by oligodendrocyte progenitor cell differentiation (*36*, *37*).

For the edge-level connectivity mvGWAS, functional annotation identified 1464 unique genes (table S17 and fig. S10). One hundred thirty-five of 211 lead SNPs had at least one eQTL annotation or chromatin interaction. For example, rs13084442 on 3q26.31 (multivariate *z* = 6.34, *P* = 2.32 × 10^−10^) is an eQTL (*38*) of *TNIK*, a gene associated with neurogenesis and intellectual disability (*39*). Similarly, the SNP rs28413051 on 4q31.23 (multivariate *z* = 6.28, *P* = 3.47 × 10^−10^) is an eQTL of *DCLK2* that is important for axon growth cone formation and neural migration (*40*) and is also within a region interacting with the promoter of *DCLK2* in neural progenitor cells (*36*). As a further example, allele C of rs13107325 on 4q24 (multivariate *z* = 5.77, *P* = 7.99 × 10^−9^ in the node-level connectivity mvGWAS and multivariate *z* = 8.74, *P* = 2.37 × 10^−18^ in the edge-level connectivity mvGWAS) is a missense coding variant in the gene *SLC39A8* that showed a high combined annotation-dependent depletion score of 23.1, which indicates that this SNP is deleterious (its frequency was 7.01%). The same SNP has been associated with white matter microstructure integrity (*20*), schizophrenia (*41*), and children’s behavioral problems (*42*).

### Gene-based association analysis and gene set enrichment analysis for the brain’s structural connectome

**Fig. 3.**
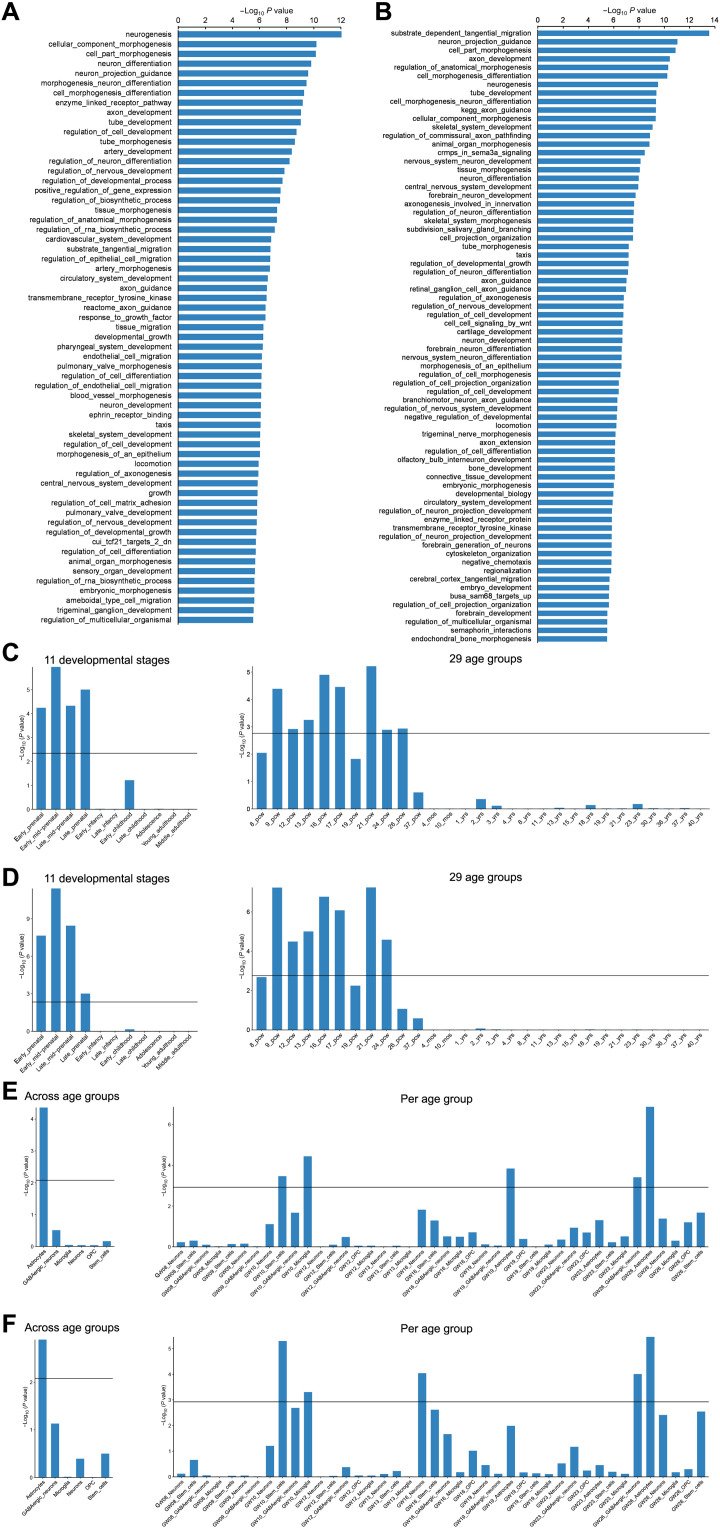
Genes associated with variation in the adult white matter connectome are enriched for specific neurodevelopmental roles. (**A**) Sixty-one functionally defined gene sets showed significant enrichment of association with node-level connectivity. (**B**) Seventy-two functionally defined gene sets showed significant enrichment of association with edge-level connectivity. (**C** and **D**) On the basis of BrainSpan data from 11 life-span stages or 29 age groups, genes associated with variation in (C) adult node-level connectivity and (D) adult edge-level connectivity show up-regulation in the human brain prenatally. (**E** and **F**) On the basis of single-cell gene expression data from the prenatal brain, genes associated with variation in (E) adult node-level connectivity show up-regulation in astrocytes when considering all prenatal age groups combined and in stem cells and microglia at 10 gestational weeks (GW), astrocytes at 19 GW, and GABAergic neurons and astrocytes at 26 GW when breaking down by developmental stages, and similarly, genes associated with variation in (F) adult edge-level connectivity show up-regulation in astrocytes when considering all prenatal age groups combined and in stem cells and microglia at 10 GW, neurons at 16 GW, and GABAergic neurons and astrocytes at 26 GW when breaking down by developmental stages. (C to F) Black lines indicate the significance threshold *P* < 0.05 after Bonferroni correction within each analysis. PCW, postconceptional weeks.

We used MAGMA (Multi-marker Analysis of GenoMic Annotation) (*23*) to perform gene-based association analysis, which combines the mvGWAS evidence for association at each SNP within a given gene while controlling for LD. For node-level connectivities, we identified 296 significant genes with *P* < 0.05 (after Bonferroni correction for testing 20,146 genes and two sets of mvGWAS results) (table S18 and fig. S11), 237 of which overlapped with those annotated by at least one of the three strategies used above (i.e., physical location, eQTL annotation, or chromatin interaction). The gene-based *P* values were then used as input to perform gene-set enrichment analysis, in relation to 15,488 previously defined functional sets within the MSigDB database (*43*). Sixty-one gene sets showed significant enrichment (Bonferroni adjusted *P* < 0.05 for testing 15,488 sets; Fig. 3A and table S19), which mainly implicated neurodevelopmental processes, such as “go_neurogenesis” (β = 0.18, *P* = 5.53 × 10^−13^; the most significant set), “go_neuron_differentiation” (β = 0.18, *P* = 1.55 × 10^−10^), and “go_cell_morphogenesis_involved_in_neuron_differentiation” (β = 0.25, *P* = 3.39 × 10^−10^).

For edge-level connectivities, we identified 561 genes with significant gene-based association (Bonferroni-corrected *P* < 0.05 for testing 20,146 genes and two sets of mvGWAS results), 444 of which overlapped with genes mapped through physical location, eQTL annotation, or chromatin interaction (table S20 and fig. S11). Seventy-two gene sets were significant after Bonferroni correction for 15,488 sets (Fig. 3B and table S21), related especially to neural migration and the development of neural projections, such as “go_substrate_dependent_cerebral_cortex_tangential_migration” (β = 3.98, *P* = 2.61 × 10^−14^; the most significant set), “go_neuron_projection_guidance” (β = 0.41, *P* = 8.59 × 10^−12^), and “go_axon_development” (β = 0.29, *P* = 3.45 × 10^−11^).

We tested our genome-wide, gene-based *P* values with respect to human brain gene expression data from the BrainSpan database (*44*), grouped according to 11 life-span stages or 29 different age groups. Genes associated with node-level connectivity showed up-regulation on average across much of the prenatal period, ranging from early (β = 0.04, *P* = 5.84 × 10^−5^) to late (β = 0.08, *P* = 1.01 × 10^−5^) prenatal stages or from 9 (β = 0.002, *P* = 4.15 × 10^−5^) to 26 (β = 0.003, *P* = 1.18 × 10^−3^) postconceptional weeks (Bonferroni-corrected *P* < 0.05; Fig. 3C and table S22). Similarly, genes associated with edge-level connectivities showed up-regulation on average during early (β = 0.06, *P* = 2.35 × 10^−8^) to late (β = 0.06, *P* = 1.01 × 10^−3^) prenatal stages or from 9 (β = 0.003, *P* = 5.92 × 10^−8^) to 24 (β = 0.003, *P* = 2.67 × 10^−5^) postconceptional weeks (Bonferroni-corrected *P* < 0.05; Fig. 3D and table S23).

We also examined our genome-wide, gene-based association *P* values with respect to two independent single-cell gene expression datasets derived from human prefrontal cortex samples of different ages (GSE104276) (*45*). Combining across age groups, average up-regulation was observed in astrocytes for genes associated with both node-level connectivity (β = 0.05, *P* = 4.34 × 10^−5^) and edge-level connectivity (β = 0.04, *P* = 1.27 × 10^−3^) (Bonferroni-corrected *P* < 0.05; Fig. 3, E and F, and tables S24 and S25). Breaking down by age, genes associated with node-level connectivity were up-regulated on average in microglia (β = 0.02, *P* = 3.72 × 10^−5^) and stem cells (β = 0.05, *P* = 3.51 × 10^−4^) at 10 gestational weeks of age (GW), astrocytes at 19 GW (β = 0.02, *P* = 1.48 × 10^−4^) and 26 GW (β = 0.05, *P* = 1.35 × 10^−7^), and GABAergic neurons at 26 GW (β = 0.04, *P* = 3.97 × 10^−4^) (Fig. 3E and table S24). Similarly, genes associated with edge-level connectivities showed up-regulation on average in microglia (β = 0.02, *P* = 5.07 × 10^−4^) and stem cells (β = 0.06, *P* = 5.18 × 10^−6^) at 10 GW, neurons at 16 GW (β = 0.06, *P* = 9.32 × 10^−5^), and astrocytes (β = 0.05, *P* = 3.50 × 10^−6^) and GABAergic neurons at 26 GW (β = 0.05, *P* = 1.01 × 10^−4^; Fig. 3F and table S25).

### Genetics of left-hemisphere language network connectivity

**Fig. 4.**
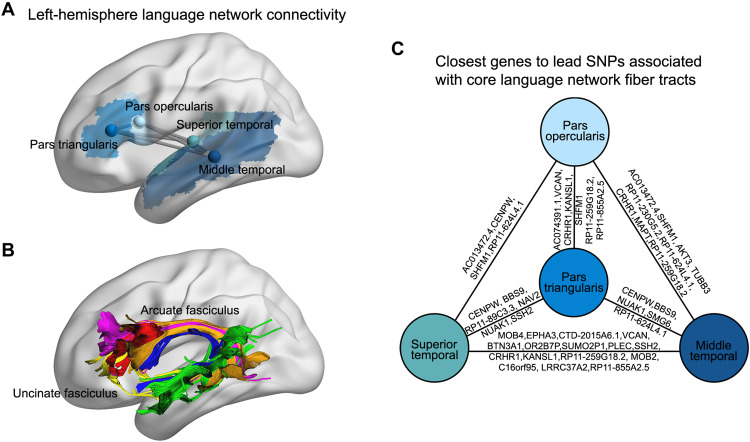
Genetics of left-hemisphere language network connectivity. (**A**) Four regions with core functions in the left-hemisphere language network, encompassing the classically defined Broca’s (frontal lobe) and Wernicke’s (temporal lobe) areas. Also shown are the six edges connecting these four regions when considered as network nodes. (**B**) Visualization of the six edges in an example individual, with red representing connections between the pars opercularis and pars triangularis, green representing connections between the middle temporal and superior temporal cortex, gold representing connections between the pars opercularis and middle temporal cortex, blue representing connections between the pars opercularis and superior temporal cortex, purple representing connections between the pars triangularis and middle temporal cortex, and yellow representing connections between pars triangularis and superior temporal cortex. (**C**) The closest genes to independent lead SNPs from the brain-wide mvGWAS of edge-level connectivity, which showed significant association with at least one of the six left-hemisphere language network edges (Bonferroni correction at 0.05; table S26).

To illustrate how the brain-wide mvGWAS results can be queried in relation to any specific brain network of interest, we selected four left-hemisphere regions that correspond to a network that is reliably activated by sentence-level language tasks in a left-lateralized manner in the majority of people and across languages (*46*), i.e., the opercular and triangular parts of inferior frontal cortex (Broca’s region) and the superior and middle temporal cortex (including the Wernicke’s region; Fig. 4). These four nodes are linked by six edges with heritabilities ranging from 7.3 to 17.1% (table S3), which together correspond well to the arcuate fasciculus and also probably include streamlines via the uncinate fasciculus—two prominent fiber tracts involved in language (Fig. 4) (*47*). Of the 211 lead SNPs from our brain-wide mvGWAS of edge-level connectivity, 31 were significantly associated with at least one of these six edges according to the edge-specific *z* scores derived from MOSTest (Bonferroni correction at 0.05; table S26). For example, rs12636275 on 3p11.1 is located within an intron of *EPHA3*, a gene that encodes an ephrin receptor subunit that regulates the formation of axon projection maps (*48*), and has also been associated with functional connectivity between language-related regions (*49*). As another example, rs7580864 on 2q33.1 is an eQTL of *PLCL1* that is implicated in autism (*50*), a neurodevelopmental disorder that often affects language and social skills. Other positional candidate genes based on the 31 SNPs include *CRHR1*, encoding corticotropin releasing hormone receptor 1, and *CENPW* (centromere protein W) involved in chromosome maintenance and the cell cycle (Fig. 4 and table S26).

### Multivariate associations of the structural connectome with polygenic scores for brain disorders and behavioral traits

For each of the 30,810 individuals in our study sample, we calculated polygenic scores (*51*) for various brain disorders or behavioral traits that have shown associations with white matter variation, using previously published GWAS summary statistics: schizophrenia (*10*, *52*–*54*), bipolar disorder (*55*, *56*), autism (*10*, *53*, *57*, *58*), attention-deficit/hyperactivity disorder (*59*, *60*), left-handedness (*61*, *62*), Alzheimer’s disease (*63*, *64*), amyotrophic lateral sclerosis (*65*, *66*), and epilepsy (*67*, *68*) (Materials and Methods). There were 18 significant partial correlations (i.e., adjusted for demographic and technical covariates; see Materials and Methods) between different pairs of these polygenic scores across individuals (Bonferroni-corrected *P* < 0.05): 16 correlations were positive, with the highest between polygenic scores for schizophrenia and bipolar disorder (*r* = 0.36, *P* < 1 × 10^−200^) and between attention-deficit/hyperactivity disorder and autism (*r* = 0.33, *P* < 1 × 10^−200^), while 2 were negative, between polygenic scores for amyotrophic lateral sclerosis and bipolar disorder (*r* = −0.03, *P* = 2.26 × 10^−6^) and amyotrophic lateral sclerosis and autism (*r* = −0.03, *P* = 8.81 × 10^−6^; table S27 and fig. S12).

Separately, for each of these polygenic scores, we used canonical correlation analysis to investigate their multivariate associations with the 90 heritable node-level connectivity measures across the 30,810 individuals. All canonical correlations were highly significant: schizophrenia: *r* = 0.07, *P* = 8.98 × 10^−34^; bipolar disorder: *r* = 0.07, *P* = 1.53 × 10^−35^; autism: *r* = 0.06, *P* = 7.87 × 10^−24^; attention-deficit/hyperactivity disorder: *r* = 0.08, *P* = 7.84 × 10^−44^; left-handedness: *r* = 0.07, *P* = 1.74 × 10^−31^; Alzheimer’s disease: *r* = 0.07, *P* = 4.14 × 10^−33^; amyotrophic lateral sclerosis: *r* = 0.06, *P* = 1.29 × 10^−25^; epilepsy: *r* = 0.05, *P* = 1.49 × 10^−20^. Therefore, polygenic dispositions to these various disorders or behavioral traits in the general population are partly reflected in the brain’s white matter connectivity.

**Fig. 5.**
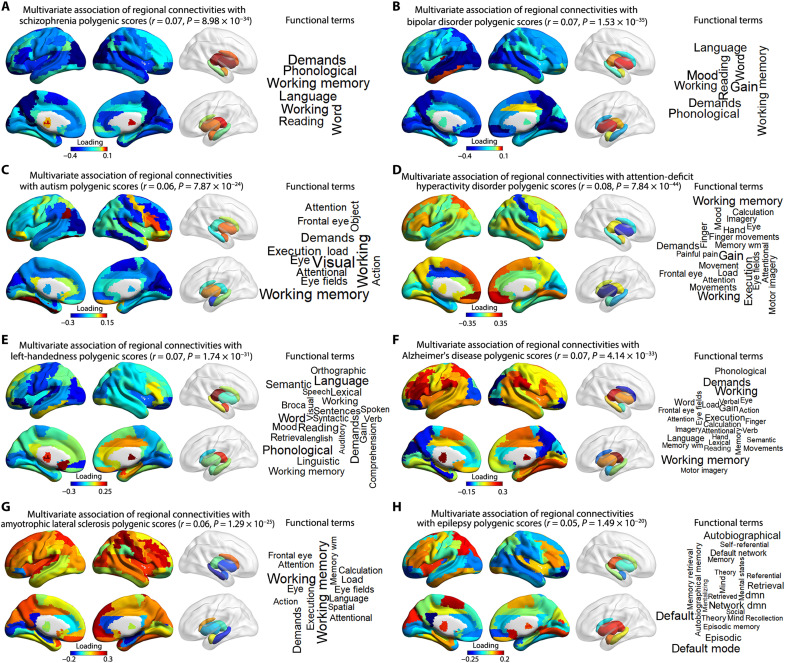
Polygenic dispositions to various brain-related disorders or behavioral traits show multivariate associations with regional (node-level) white matter connectivities in 30,810 participants. Loadings are shown from canonical correlation analyses that indicate the extent and direction to which each node-level connectivity is associated with polygenic scores for (**A**) schizophrenia, (**B**) bipolar disorder, (**C**) autism, (**D**) attention-deficit/hyperactivity disorder, (**E**) left-handedness, (**F**) Alzheimer’s disease, (**G**) amyotrophic lateral sclerosis, and (**H**) epilepsy. A positive loading (red) indicates a higher–node-level connectivity associated with increased polygenic disposition for a given disorder/behavioral trait, while a negative loading (blue) represents a lower–node-level connectivity associated with increased polygenic disposition for a given disorder/behavioral trait. Word clouds represent functions associated with the map of regions (nodes) showing the strongest loadings (|*r*| > 0.2) for each polygenic score. Functions were assigned using large-scale meta-analyzed functional neuroimaging data (Materials and Methods). The font sizes in the word clouds represent correlation magnitudes between the meta-analyzed functional maps for those terms and the coactivation map for the set of regions associated with each polygenic score. See table S30 for the correlation coefficients. wm, working memory; dmn, default-mode network.

Canonical correlation analyses yielded loadings for each node-level connectivity measure, reflecting the extent and direction of each measure’s association with polygenic disposition for a given disorder/behavioral trait. For psychiatric disorders, the majority of loadings were negative, i.e., increased polygenic risk for these disorders was more often associated with reduced than increased connectivity across brain regions (Fig. 5 and table S28). This was especially marked for polygenic risks for schizophrenia (85 regions with negative loadings, 5 regions with positive loadings), bipolar disorder (81 negative, 9 positive), and autism (64 negative, 26 positive). Polygenic disposition to left-handedness was also associated with more reduced node-level connectivities (62 negative loadings) than increased node-level connectivities (28 positive loadings). In contrast, increased polygenic risk for Alzheimer’s disease was associated with increased white matter connectivity for a majority of brain regions (62 of 90) in the U.K. Biobank data, even while some known regions of disorder pathology showed decreased connectivity, such as medial temporal cortex (*69*). (These results remained stable when excluding the *APOE* locus that is known to have a substantial individual effect on Alzheimer’s disease risk; see Materials and Methods and table S29). Similar observations were made for polygenic risk for amyotrophic lateral sclerosis, where 74 of 90 regions showed positive loadings (Fig. 5 and table S28).

For each polygenic score, we identified the specific node-level connectivities that showed the strongest loadings in canonical correlation analyses, i.e., brain regions with loadings of >0.2 or <−0.2. These regions were used to create a single brain mask for each polygenic score, which was then used to query the Neurosynth database of 14,371 functional brain imaging studies (*70*). In this process, a brain-wide coactivation map was generated for each mask, based on all functional maps in the database, and these were then correlated with cognitive and behavioral term-specific maps derived from the studies included in the database (*70*).

For example, the mask for schizophrenia polygenic risk comprised 32 regions showing the strongest associations with white matter connectivity, distributed in the bilateral temporal, dorsoventral, and posterior cingulate cortex (Fig. 5A and table S28), and there were seven functional term-based correlations of >0.2 with the corresponding coactivation map for these regions (Fig. 5A, fig. S13, and table S30), including “working memory” and “language.” This suggests that polygenic disposition to schizophrenia influences the connectivity of brain regions especially involved in working memory and language (see Discussion). The mask for bipolar disorder polygenic risk comprised 30 regions, including temporal, medial frontal, superior parietal, and visual cortex, as well as hippocampus and caudate, and these regions together received functional annotations of “mood,” working memory, and language-related processes (Fig. 5B, fig. S13, and tables S28 and S30). Polygenic risk for autism was mainly associated with white matter connectivity of the right dorsolateral prefrontal, right temporal, right sensorimotor, and bilateral visual cortex, as well as the left amygdala, and these regions were annotated with visual, working memory, executive, and attention functions (Fig. 5C, fig. S13, and tables S28 and S30). Polygenic disposition to left-handedness was associated with node-level connectivity of Broca’s area, left superior temporal cortex, left medial prefrontal and left visual cortex, and right thalamus, functionally annotated with language-related cognitive functions (Fig. 5E, fig. S13, and tables S28 and S30). See Fig. 5, fig. S13, and tables S28 and S30 for the equivalent maps and functional annotations for all disorder/trait polygenic scores. The polygenic risks for bipolar disorder and schizophrenia had the most similar brain maps, in terms of node-level structural connectivity associated with each of these polygenic risks (*r* = 0.56 between the loadings for these two polygenic scores, across the 90 nodes; fig. S14 and table S31).

## DISCUSSION

This large-scale mapping study used white matter tractography and multivariate analysis to characterize the contributions of common genetic variants to individual differences in structural connectivity of the adult human brain. Multivariate associations between structural connectivity and polygenic dispositions to brain-related disorders or behavioral traits were also characterized and described in terms of functional activations of the implicated brain regions. Together, these various analyses in over 30,000 individuals from the general population linked multiple levels of biological organization: from genes and cell types through developmental stages to adult brain structure and function, behavior, and individual differences, implicating hundreds of genomic loci that had not previously been associated with human brain measures.

Different brain regions are interconnected through white matter nerve fibers; this fundamental property subserves functional networks involved in cognition and behavior. In over 30,000 adults from the general population, we found that interindividual variation in white matter connectivity is especially influenced by genes that are (i) active in the prenatal developing brain; (ii) up-regulated in stem cells, astrocytes, microglia, and neurons of the embryonic and fetal brain; and (iii) involved in neurodevelopmental processes including neural migration, neural projection guidance, and axon development. A likely neurodevelopmental origin of much interindividual variation of adult white matter connectivity is consistent with findings from large-scale imaging genetic studies of other aspects of brain structural and functional variation (*10*, *21*, *58*). These statistical enrichment findings serve as a strong biological validation of our mvGWAS findings, as there was no reason for such clearly relevant functional enrichment to occur by chance in relation to brain white matter tracts.

Astrocytes are the largest class of brain glial cells with a range of known functions, including neuronal homeostasis and survival, regulation of synaptogenesis, and synaptic transmission (*71*). Less well known is that during neurodevelopment, astrocytes can express positional guidance cues, such as semaphorin 3a, that are required for neuronal circuit formation, through mediating the attraction or repulsion of the growth cone at the axonal tip (*72*). In our gene-based association analysis, *SEMA3A* was the most significantly associated individual gene with edge-level connectivity in the whole genome. Together, our data suggest that the formation of fiber tracts in the developing human brain may be affected substantially by positional cues provided by astrocytes, in addition to neurons.

As regard microglia, these phagocytic cells not only are well known for immune functions but also help to remove dying neurons and prune synapses, as well as modulate neuronal activity (*73*). Less is known of their roles during development, but embryonic microglia are unevenly distributed in the brain and associate with developing axons, which again suggests roles in regulating axonal growth and positional guidance (*74*). Mouse brains without microglia, or with immune activated microglia, show abnormal dopaminergic axon outgrowth (*75*), while disruption of microglial function or depletion of microglia results in a failure of growing axons to adhere and form bundles in the corpus callosum, the largest fiber tract of the brain (*76*). Our data support such observations, through showing that genes up-regulated in microglia in the embryonic human brain are enriched for variants that associate with individual differences in adult white matter connectivity. Further research on the roles of astrocytes and microglia in fiber tract development is therefore warranted.

While our results point especially to genes involved in neurodevelopment, it is also likely that some genetic effects on white matter connectivity act later in life. For example, astrocytes and microglia may affect the maintenance and aging of brain fiber tracts during adulthood, with implications for brain disorders and possibly suggesting therapeutic targets. We mapped the multivariate associations of polygenic scores for various brain-related disorders and behavioral traits with regional white matter connectivities and annotated the resulting brain maps using meta-analyzed functional imaging data. Some maps and their annotations were consistent with the symptomatology of the traits in question—for example, polygenic disposition to bipolar disorder was associated with white matter connectivity of brain regions prominently involved in mood, while polygenic dispositions to attention-deficit/hyperactivity disorder or autism were associated with the connectivity of regions important for executive functions. Polygenic scores for left-handedness and for schizophrenia were associated with the connectivity of language-related regions, consistent with altered left-hemisphere functional dominance for language in both of these traits (*12*, *77*) and a phenotypic association between them (*78*). Polygenic scores for left-handedness and schizophrenia have also been associated with altered structural asymmetry of gray matter in language-related regions (*53*, *61*).

Regarding genetic risks for neurological disorders, polygenic scores for Alzheimer’s disease and amyotrophic lateral sclerosis were associated with the connectivity of regions important for working memory, while polygenic scores for epilepsy were associated with connectivity of the default mode network, a set of brain regions involved in internally initiated thoughts and semantic and episodic memory (*79*). Previous analysis of white matter tracts in Alzheimer’s disease has indicated a broad-based reduction of connectivity (*80*), so it was unexpected that the majority of brain regions in the U.K. Biobank adult population dataset showed increased connectivity with higher polygenic risk for this disorder, even while some core regions of pathology showed decreased connectivity as expected. A similarly notable pattern was seen for amyotrophic lateral sclerosis, where increased polygenic risk was associated with increased structural connectivity for a majority of brain regions. It may be that increased connectivity of some regions occurs as a compensatory reconfiguration in response to decreased connectivity of others, or at least that white matter connectivity is relatively spared while cortical gray matter is reduced during aging of those at higher polygenic risk.

The brain-wide mvGWAS approach that we used provided high statistical power to detect relevant genomic loci, compared to a mass univariate approach (*16*). At the same time, the multivariate results could be queried post hoc to identify loci associated with particular edge-level connectivities of interest. We illustrated this by querying the results with respect to six connections linking four core regions of the left-hemisphere language network, together approximating to Broca’s and Wernicke’s classically defined functional areas (*46*). As expected, these connections together formed an overall feature that closely resembles the arcuate fasciculus plus some connections running through the uncinate fasciculus (Fig. 4), the two major language-related tracts (*47*, *81*). Thirty-one implicated loci included the *EPHA3* locus, encoding an ephrin receptor subunit that acts as a positional guidance cue for the formation of axon projection maps and has also been associated with functional connectivity between regional components of the language network that are especially involved in semantics (*49*). This is therefore a concordant genetic finding with respect to both structural and functional connectivity of the human brain’s language network.

In this study, we used deterministic tractography which we found to be computationally feasible in more than 30,000 individuals (and which took roughly 4 months of processing on a cluster server). An alternative approach, probabilistic tractography, is generally more demanding in terms of run time and storage requirements but can have advantages, especially as it permits modeling of multiple tract orientations per voxel and therefore allows for crossing fibers (*82*). However, it has been reported that deterministic tractography tends to have a lower likelihood of generating false-positive connections than probabilistic approaches (*83*). This is important because false positives can be more detrimental to the correct calculation of network measures than false negatives (*83*). Similarly, Sarwar *et al.* (*84*) assessed the performance across tractography models and reported that deterministic tractography yielded the most accurate connectome reconstructions, especially when omitting connections with the fewest number of streamlines. Accordingly, we only included a connection in our structural connectivity matrix when it was detected in at least 80% of participants. Applying this threshold removed 3058 weak or spurious connections from our study, leaving 947 for further analysis, of which 851 showed significant heritability and were taken forward into mvGWAS analysis. This threshold has been found to be suitable for white matter network property analysis, as it provides a balance between the elimination of false-positive connections and creating false-negative connections (*85*). Anatomical connectivity constructed by deterministic tractography has been well confirmed by microdissection in the postmortem human brain (*86*), indicating robustness and reliability of this approach (*87*). In addition, deterministic tractography has been widely used to construct white matter connectivity patterns in previous diffusion MRI studies, which investigated structural characteristics during neurodevelopment, aging, and in brain disorders (*4*, *88*, *89*).

It has been reported that tract specificity can be lost through bottlenecks such as the corpus callosum when applying DTI-based tractography (*90*), although other studies have reported that deterministic tractography can achieve successful reconstruction of interhemispheric connections via the corpus callosum (*91*, *92*). In addition, interhemispheric connectivity via the corpus callosum that was reconstructed from conventional diffusion imaging data has been supported by high angular resolution diffusion imaging and also by postmortem examination of white matter anatomy (*93*–*95*). We found the average heritability of interhemispheric connections to be only slightly lower than for intrahemispheric connections, which suggests that the interhemispheric connections were measured reliably enough to contribute to the identification of genetic effects.

We measured structural connectivity linking pairs of regions defined according to parcellation at the macroscopic level, using the AAL (Automatic Anatomical Labeling) atlas (*13*). This atlas was created by manual neuroanatomical delineation on the basis of high-resolution structural imaging. Previous studies have found that this atlas, in combination with DTI data, reliably indexes structural connectivity (*96*–*98*). In addition, the AAL atlas in combination with deterministic tractography has been applied before in a study of vascular burden and cognitive ability in the U.K. Biobank dataset (*99*), which showed that this protocol is suitable and practical given the large size and scanning resolution of this dataset. Furthermore, AAL atlas regions are defined in volume space and are therefore consistent to apply across cortical and subcortical structures (unlike cortical surface–based segmentations), which was a goal of the present study. Tractography using volume-based atlases, such as AAL, has also been shown to capture variation arising from cortical thickness or bundle shape better than surface-based atlases (*100*, *101*). The AAL labeling system integrates anatomical features from sulcal and gyral geometry, while the relatively large regions help to overcome variability arising during spatial registration and normalization of brain images from different individuals. While it would be informative to apply different atlases in future studies, it is likely that the percentage of interregional connections not detected (i.e., with zero streamlines) would increase with a more fine-grained atlas having more, smaller parcellations.

This study had some limitations: (i) We maximized our statistical power for GWAS using the available data as one large discovery sample, but this did not permit a discovery-replication design. Nonetheless, ultimately, the total combined analysis in the largest available sample is the most representative of the available evidence for association. As mentioned earlier in this section, the various enrichment analyses indicated biological validity of the GWAS findings. It has been argued that discovery-replication designs have less utility in the current era of Biobank-scale genetic studies than they used to and that other forms of validation such as biological enrichment should be given increased weight in interpretation (*102*). (ii) This was a large-scale observational mapping study, which meant that many of the analyses were screen-based and descriptive. Science proceeds through a combination of observation and hypothesis testing—this study incorporated both to varying degrees. Some of the biological observations were notable and informative, for example, the likely involvements of microglia and astrocytes in affecting white matter tracts during embryonic and fetal development, which should now be studied more extensively in animal models. (iii) This study did not consider rare genetic variants (with population frequencies below 1%). Future analysis of the U.K. Biobank’s exome and genome sequence data in relation to white matter connectivity may reveal further genes and suggest additional mechanisms, cell types, and life-span stages in affecting interindividual variation.

In summary, we used large-scale analysis to chart the white matter connectivity of the human brain, its multivariate genetic architecture, and its associations with polygenic dispositions to brain-related disorders and behavioral traits. The analyses implicated specific genomic loci, genes, pathways, cell types, developmental stages, brain regions, fiber tracts, and cognitive functions, thus integrating multiple levels of analysis and suggesting a range of future research directions at each of these levels.

## MATERIALS AND METHODS

### Sample quality control

This study was conducted under U.K. Biobank application 16066, with C.F. as principal investigator. The U.K. Biobank received ethical approval from the National Research Ethics Service Committee North West-Haydock (reference 11/NW/0382), and all of their procedures were performed in accordance with the World Medical Association guidelines (*103*). Written informed consent was provided by all of the enrolled participants. We used the dMRI (diffusion MRI) data released in February 2020, together with the genome-wide genotyping array data. For individuals with available dMRI and genotype data, we first excluded participants with a mismatch of their self-reported and genetically inferred sex, with putative sex chromosome aneuploidies, or who were outliers according to heterozygosity (principle component corrected heterozygosity > 0.19) and genotype missingness (missing rate > 0.05) as computed by Bycroft *et al.* (*104*). To ensure a high degree of genetic homogeneity, analysis was limited to participants with white British ancestry, which was defined by Bycroft *et al.* (*104*), using a combination of self-report and cluster analysis based on the first six principal components that capture genetic ancestry. We also randomly excluded one participant from each pair with a kinship coefficient of >0.0442, as calculated by Bycroft *et al.* (*104*). All of these metrics are available within U.K. Biobank data category 263 or 100313. Our inclusion procedure lastly resulted in 30,810 participants, with a mean age of 63.84 years (range, 45 to 81 years), 14,636 were male and 16,174 were female.

### Genetic quality control

We downloaded imputed SNP and insertion/deletion genotype data from the U.K. Biobank (i.e., v3 imputed data released in March 2018; U.K. Biobank data category 263 and data field 22828). QCTOOL (v.2.0.6) and PLINK v2.0 (*105*) were used to perform genotype quality control. Specifically, we excluded variants with a minor allele frequency of <1%, a Hardy-Weinberg equilibrium test *P* < 1 × 10^−7^, and an imputation INFO score of <0.7 (a measure of genotype imputation confidence), followed by removing multiallelic variants that cannot be handled by many programs used in genetic-related analyses. This pipeline lastly yielded 9,803,735 biallelic variants.

### Diffusion MRI-based tractography

Diffusion MRI data were acquired from Siemens Skyra 3 T scanners running protocol VD13A SP4, with a standard Siemens 32-channel RF receive head coil (*106*). We downloaded the quality-controlled dMRI data that were preprocessed by the U.K. Biobank brain imaging team (*106*, *107*) (U.K. Biobank data field: 20250, first imaging visit). The preprocessing pipeline included corrections for eddy currents, head motion, outlier slices, and gradient distortion. We did not make use of imaging-derived phenotypes released by the U.K. Biobank team, such as FA (fractional anisotropy) and mean diffusivity (microstructural measures). Rather, we used the quality-controlled dMRI data to perform tractography in volume space in each individual, which generated three-dimensional curves that characterize white matter fiber tracts. Briefly, diffusion tensors were modeled to generate an FA image in native diffusion space, which was used for deterministic diffusion tensor tractography using MRtrix3 (*108*). Streamlines were seeded on a 0.5-mm grid for every voxel with an FA of 0.15 and propagated in 0.5-mm steps using fourth-order Runge-Kutta integration. Tractography was terminated if the streamline length was <20 or >250 mm, if it turned an angle of >45°, or reached a voxel with an FA of <0.15. These parameters were consistent with a previous study exploring the structural network correlates of cognitive performance using the U.K. Biobank dataset (*99*). Tens of thousands of streamlines were generated to reconstruct the white matter connectivity matrix of each individual on the basis of the Automated Anatomical Labelling atlas (*13*) comprising a total of 90 regions encompassing cortical and subcortical structures (45 regions per hemisphere). This deterministic tractography process took roughly 16 weeks on six cluster server nodes running in parallel.

From the streamline data, we computed the mean lengths of all interregional connections reconstructed by the deterministic tractography, using the “scale_length” option of the “tck2connectome” function in the MRtrix3 toolbox (*109*).

### Network construction and analysis

Describing the structural network of each participant requires the definition of network nodes and edges. In this study, the network nodes corresponded to the 90 regions of the Automated Anatomical Labeling atlas, a three-dimensional volume-based parcellation scheme (*13*). The labeling system integrates detailed anatomical features from sulcal and gyral geometry, reducing anatomical variability that can arise from spatial registration and normalization of brain images taken from different individuals (*13*). For each participant, the T1 images (U.K. Biobank data field: 20252, first imaging visit) were nonlinearly transformed into the ICBM152 T1 template in the MNI (Montreal Neurological Institute) space to generate the transformation matrix (*110*). Inverse transformation was used to warp the Automated Anatomical Labeling atlas (*13*) from the MNI volume space to native volume space. Discrete labeling values were preserved using a nearest-neighbor interpolation method (*110*). Two nodes were considered connected if they were joined by the end points of at least one reconstructed streamline. Separately, for each individual in the dataset, network edges were computed by the number of streamlines connecting a given pair of regions, divided by the volume of the two regions, because regions with larger volumes tend to have more streamlines connecting to them. This is a common approach in studies of white matter networks (*111*–*113*). We only included edges that were detected in at least 80% of participants, which removed 3058 weak or spurious connections from our study. This yielded a zero-diagonal symmetrical 90 × 90 undirected connectivity matrix for each participant, in which 947 edges were retained. The node-level connectivity of a region was then defined as the sum of all existing volume-weighted edges between that node and all other nodes in the network, reflecting the total connectivity of that node within the overall network.

Rank-based inverse normalization across individuals was performed on each network measure and regression on age (U.K. Biobank field: 21003), nonlinear age [i.e., (age-mean_age)^2^], assessment center (U.K. Biobank data field: 54), genotype measurement batch (data field: 22000), and sex (data field: 31). Residuals were then further regressed on the first 10 genetic principal components that capture population genetic diversity (U.K. Biobank field: 22009) (*104*), followed by rank-based inverse normalization of the residuals once more, and visual inspection of their distributions to confirm normality. The normalized, transformed measures were used for subsequent genetic analyses.

### SNP-based heritability

We constructed a genetic relationship matrix using 9,516,306 variants on the autosomes with minor allele frequencies of >1%, an INFO score of >0.7, and Hardy-Weinberg equilibrium *P* > 1 × 10^−7^, using GCTA (version 1.93.0beta) (*15*). We further excluded one random participant from each pair having a kinship coefficient higher than 0.025 (as SNP-based heritability analysis is especially sensitive to participants with higher levels of relatedness), yielding 29,027 participants for this particular analysis. Genome-based restricted maximum likelihood analyses were then performed to estimate the SNP-based heritability for each normalized structural network measure, again using GCTA (*15*). Bonferroni correction was applied separately for each type of network measure to identify those that were significantly heritable at adjusted *P* < 0.05: 90 node-level connectivities and 851 edge-level connectivities.

### Reliability of heritable network measures

For our main analysis of the 30,810 individuals (above), we used data from the first scanning visit at a U.K. Biobank assessment center. One thousand five of these individuals had also undergone brain scans (T1 structural and DTI) on a subsequent, separate occasion, from 733 to 974 days after their first scan. To examine the reliability of significantly heritable brain network measures we re-ran deterministic tractography on the “second scan” data from these 1005 individuals, with the same set of parameters and quality filters as the primary analysis, and recomputed the same edge-wise connectivity metrics as in the primary analysis. Each edge measure was then linearly adjusted for the same covariates as the main analysis, and rank-based inverse normalization was applied as in the main analysis. The adjusted, normalized values from the first and second scans were then used to compute the ICC for each heritable network measure, to evaluate reliability (*114*). ICC was calculated by the following formula

$$\mathrm{ICC}=\frac{\mathrm{BMS}-\mathrm{WMS}}{\mathrm{BMS}+(m-1)\mathrm{WMS}}$$

where BMS represents the between-individual mean square, WMS represents the within-individual mean square, and *m* indicates the number of repeat measures (here, *m* = 2).

### Multivariate genome-wide association analysis

A total of 9,803,735 biallelic variants were used for mvGWAS analysis, spanning all autosomes and chromosome X. The sample size for mvGWAS was 30,810 (see the “Sample quality control” section above). We applied the MOSTest toolbox (*16*) to perform mvGWAS analysis for the significantly heritable measures, separately for node-level connectivities and edge-level connectivities. MOSTest can leverage the distributed nature of genetic influences across hundreds of spatially distributed brain phenotypes while accounting for their covariances, which can boost statistical power to detect variant-phenotype associations (*16*). Specifically, the multivariate correlation structure is determined on randomly permuted genotype data. MOSTest calculates the Mahalanobis norm as the sum of squared decorrelated *z* values across univariate GWAS summary statistics, to integrate effects across measures into a multivariate *z* statistic for each genetic variant, and uses the gamma cumulative density function to fit an analytic form for the null distribution. This permits extrapolation of the null distribution below the *P* = 5 × 10^−8^ significance threshold without performing an unfeasible number of permutations [5 × 10^−8^ is a widely used threshold for GWAS multiple test correction in European-descent populations (*115*)]. Close matching of the null *P* value distributions from the permuted and analytic forms indicates that the method correctly controls type 1 error; this was the case for all four of our mvGWAS analyses (figs. S6 and S7). In this framework, the signs (positive or negative) of univariate *z* scores indicate the corresponding directions of effects (with respect to increasing numbers of minor alleles at a given SNP), whereas multivariate *z* scores are always positive.

### Identification of genomic loci, functional annotations, and SNP-to-gene mapping

We used FUMA (version v1.4.0) (*17*) to identify distinct genomic loci showing significant multivariate associations with brain structural connectivity and applied functional annotations, using default parameters. LD structure was applied according to the 1000 Genomes European reference panel (*116*). SNPs with genome-wide significant mvGWAS *P* < 2.5 × 10^−8^ that had LD *r*^2^ < 0.6 with any others were identified. For each of these SNPs, other SNPs that had *r*^2^ ≥ 0.6 with them were included for further annotation (see below), and independent “lead SNPs” were also defined among them as having low LD (*r*^2^ < 0.1) with any others. If LD blocks of significant SNPs were located within 250 kb of each other, then they were merged into one genomic locus. Therefore, some genomic loci could include one or more independent lead SNPs. The major histocompatibility complex region on chromosome 6 was excluded from this process by default, because of its especially complex and long-range LD structure. Functional annotation and SNP-to-gene mapping were carried out in FUMA according to previously published criteria (*58*).

### Multivariate association profiles of independently associated lead SNPs

For each SNP, MOSTest derives a *z* score for each brain measure, calculated from the *P* value of the univariate association of that SNP with each individual measure. The *z* scores give an indication of which measures contribute most to the multivariate association for a given SNP (*16*). We used the *z* scores from the mvGWAS of fiber tracts to identify lead SNPs that were significantly associated with at least one from a set of six left-hemisphere language-related fiber tracts (see Results: Genetics of left-hemisphere language connectivity). To determine significance in this context, a threshold *z* score with an unsigned magnitude of >3.56 was applied, corresponding to a *P* value of 2.37 × 10^−4^ (i.e., *P* < 0.05 after Bonferroni correction for all 211 lead SNPs from the mvGWAS of fiber tracts and considering six fiber tracts). To determine which structural connectivity measures contributed most to the multivariate associations as considered across lead SNPs, we summed the unsigned univariate *z* scores separately for each measure across all lead SNPs (separately for the mvGWAS analyses of node-level connectivities and fiber tracts).

### Gene-based association analysis

MAGMA (v1.08) (*23*), with default parameters as implemented in FUMA (SNP-wise mean model), was used to test the joint association arising from all SNPs within a given gene (including 50-kb upstream to 50-kb downstream) while accounting for LD between SNPs. SNPs were mapped to 20,146 protein-coding genes on the basis of National Center for Biotechnology Information build 37.3 gene definitions, and each gene was represented by at least one SNP. Bonferroni correction was applied for the number of genes (*P* < 0.025/20,146), separately for each mvGWAS.

### Gene-set enrichment analysis

MAGMA (v1.08), with default settings as implemented in FUMA, was used to examine the enrichment of association for predefined gene sets. This process tests whether gene-based *P* values among all 20,146 genes are lower for those genes within predefined functional sets than the rest of the genes in the genome while correcting for other gene properties such as the number of SNPs. A total of 15,488 gene sets from the MSigDB database version 7.0 (*43*) [5500 curated gene sets, 7343 gene ontology (GO) biological processes, 1644 GO molecular functions, and 1001 GO cellular components] were tested. Bonferroni correction was applied to correct for the number of gene sets (*P* < 0.05/15,488), separately for each mvGWAS.

### Cell type–specific expression analysis in developing human cortex

On the basis of a linear regression model, the CELL TYPE function of FUMA was used to test whether gene-based association *z* scores were positively associated with higher expression levels in certain cell types, based on single-cell RNA sequencing data from the developing human prefrontal cortex (GSE104276) (*45*). This dataset comprised (i) expression per cell type per age group, ranging from 8 to 26 postconceptional weeks, and (ii) expression profiles per cell type, averaged over all ages combined. Results were considered significant if the association *P* values were smaller than the relevant Bonferroni threshold for the number of cell types/age groups. Analysis was performed separately for each mvGWAS.

### Developmental stage analysis

We used MAGMA (default settings as implemented in FUMA) to examine whether lower gene-based association *P* values tended to be found for genes showing relatively higher expression in BrainSpan gene expression data (*44*) from any particular life-span stage when contrasted with all other stages, separately for 29 different age groups ranging from 8 postconceptional weeks to 40 years old, and 11 defined life-span stages from early prenatal to middle adulthood. A false discovery rate threshold of 0.05 was applied separately for each analysis. Positive β coefficients for this test indicate that genes showing more evidence for association are relatively up-regulated on average at a given life-span stage.

The Brainspan study originally collected and assigned human brain postmortem tissue samples to 1 of 31 developmental/life-span stages (*44*), but FUMA’s implementation excluded two age groups that had less than three samples each (i.e., 25 postconceptional weeks and 35 postconceptional weeks), resulting in 29 age groups being specified for this analysis by FUMA.

### Polygenic disposition to brain-related disorders or behavioral traits

We used PRS-CS (*51*) to compute polygenic scores for 30,810 U.K. Biobank individuals (see the “Sample quality control” section) for each of the following brain-related disorders or behavioral traits, using GWAS summary statistics from previously published, large-scale studies: schizophrenia (*n* = 82,315) (*52*), bipolar disorder (*n* = 51,710) (*55*), autism (*n* = 46,350) (*57*), attention-deficit/hyperactivity disorder (*n* = 55,374) (*60*), left-handedness (*n* = 306,377) (*61*), Alzheimer’s disease (*n* = 63,926) (*63*), amyotrophic lateral sclerosis (*n* = 152,268) (*66*), and epilepsy (*n* = 44,889) (*68*). None of these previous studies used U.K. Biobank data, except for the GWAS of left-handedness (*61*); however, the individuals in that GWAS were selected to be nonoverlapping and unrelated to those with brain image data from the February 2020 data release, so that none of the 30,810 U.K. Biobank individuals from the present study had been included in that GWAS. This ensured that training and target sets for polygenic score calculation were independent. PRS-CS infers posterior effect sizes of autosomal SNPs on the basis of genome-wide association summary statistics, within a high-dimensional Bayesian regression framework. We used default parameters and the recommended global effect size shrinkage parameter ϕ = 0.01, together with LD information based on the 1000 Genomes Project phase 3 European-descent reference panel (*117*). Polygenic scores were calculated using 1,097,390 SNPs for schizophrenia, 1,098,372 SNPs for bipolar disorder, 1,092,080 SNPs for autism, 1,042,054 SNPs for attention-deficit/hyperactivity disorder, 1,103,632 SNPs for left-handedness, 1,105,067 SNPs for Alzheimer’s disease, 1,085,071 SNPs for amyotrophic lateral sclerosis, and 852,343 SNPs for epilepsy (these numbers came from three-way overlaps between U.K. Biobank data, GWAS results, and 1000 Genomes data). PRS-CS has been shown to perform in a highly similar manner to other established polygenic risk algorithms, with noticeably better out-of-sample prediction than an approach based on *P* value thresholds and LD clumping (*118*, *119*).

Polygenic scores were linearly adjusted for the effects of age, nonlinear age [i.e., (age-mean_age)^2^], assessment center, genotype measurement batch, sex, and the first 10 genetic principal components that capture population genetic diversity, before performing rank-based inverse normalization (i.e., the same set of covariate effects that the brain metrics were adjusted for; see the “Network construction and analysis” section) and visual inspection of their distributions to confirm normality. The adjusted and normalized polygenic scores were used as input for subsequent analyses.

Separately, for polygenic scores for each disorder or behavioral trait, canonical correlation analysis across 30,810 participants (“canoncorr” function in MATLAB) was used to test multivariate association with the 90 heritable node-level connectivity measures (which had also been adjusted for covariates and normalized; see the “Network construction and analysis” section). This multivariate analysis identified a linear combination of the 90 node-level connectivity measures (i.e., a canonical variable) that maximally correlated with the polygenic score for a particular disorder or behavioral trait across participants. Separately, for the polygenic score of each disorder or behavioral trait, the cross-participant Pearson correlation between each node-level connectivity and the canonical variable was used as a loading, reflecting the extent and direction of the contribution that a node-level connectivity made to a particular multivariate association. We also assessed the pairwise correlations across individuals between adjusted and normalized polygenic scores for the different disorders and behavioral traits.

As the *APOE* locus is known to have a substantial effect on the risk of Alzheimer’s disease, we also recalculated polygenic scores for this disease after excluding a region from Chr19:45,116,911 to Chr19:46,318,605 (GRCh37) (*120*) around this locus and repeated the residualization, normalization, and canonical correlation analyses to check that the results stably reflected the polygenic contribution to risk.

### Functional annotation of brain regions associated most strongly with polygenic scores

From each separate canonical correlation analysis of polygenic scores and node-level connectivity, we identified the regions showing loadings of >0.2 or <−0.2, which were then used to define a single mask in standard brain space (Montreal Neurological Institute space 152) (i.e., one mask for each polygenic score). Each mask was analyzed using the “decoder” function of the Neurosynth database (http://neurosynth.org), a platform for large-scale synthesis of functional MRI data (*70*). This database defines brain-wide activation maps corresponding to specific cognitive or behavioral task terms using meta-analyzed functional activation maps. The database included 1334 term-specific activation maps corresponding to cognitive or behavioral terms from 14,371 studies. Each mask that we created was used separately as input to define a brain-wide coactivation map based on all studies in the database. The resulting coactivation maps were then correlated with each of the 1334 term-specific activation maps (*70*). We report only terms with correlation coefficients *r* > 0.2 while excluding anatomical terms, nonspecific terms (e.g., “Tasks”), and one from each pair of virtually duplicated terms (such as “Words” and “Word”). This method does not use inferential statistical testing but rather ranks terms based on the correlations between their activation maps and that of the input mask.

## Notes

View/request a protocol for this paper from *Bio-protocol*.
