# Supplementary material for: Genetic architecture of the white matter connectome of the human brain
Source: Sci Adv. 2023 Feb 17;9(7):eadd2870. doi: 10.1126/sciadv.add2870 (PMC9937579; doi:10.1126/sciadv.add2870)
Supplement: Supplementary file 1 — Figs. S1 to S14 Legends for tables S1 to S31 [file sciadv.add2870_sm.pdf]

Science Advances
AAAS

Supplementary Materials for

Genetic architecture of the white matter connectome of the human brain

Zhiqiang Sha *et al.*

Corresponding author: Clyde Francks, clyde.francks@mpi.nl

Sci. Adv. **9**, eadd2870 (2023)
DOI: 10.1126/sciadv.add2870

The PDF file includes:

Figs. S1 to S14
Legends for tables S1 to S31

Other Supplementary Material for this manuscript includes the following:

Tables S1 to S31

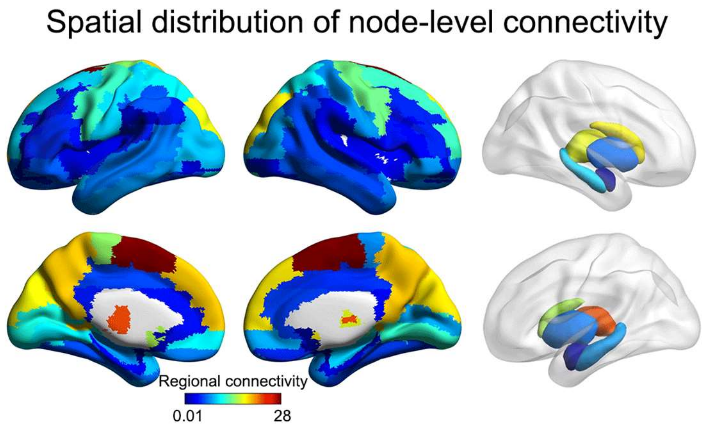

Supplementary Figure 1. Spatial distribution of node-level connectivity. The connectivity of each region of the AAL brain atlas was calculated as the sum of its white matter connections to all other regions (see Methods). Regions in orange-red colours had the highest connectivity.

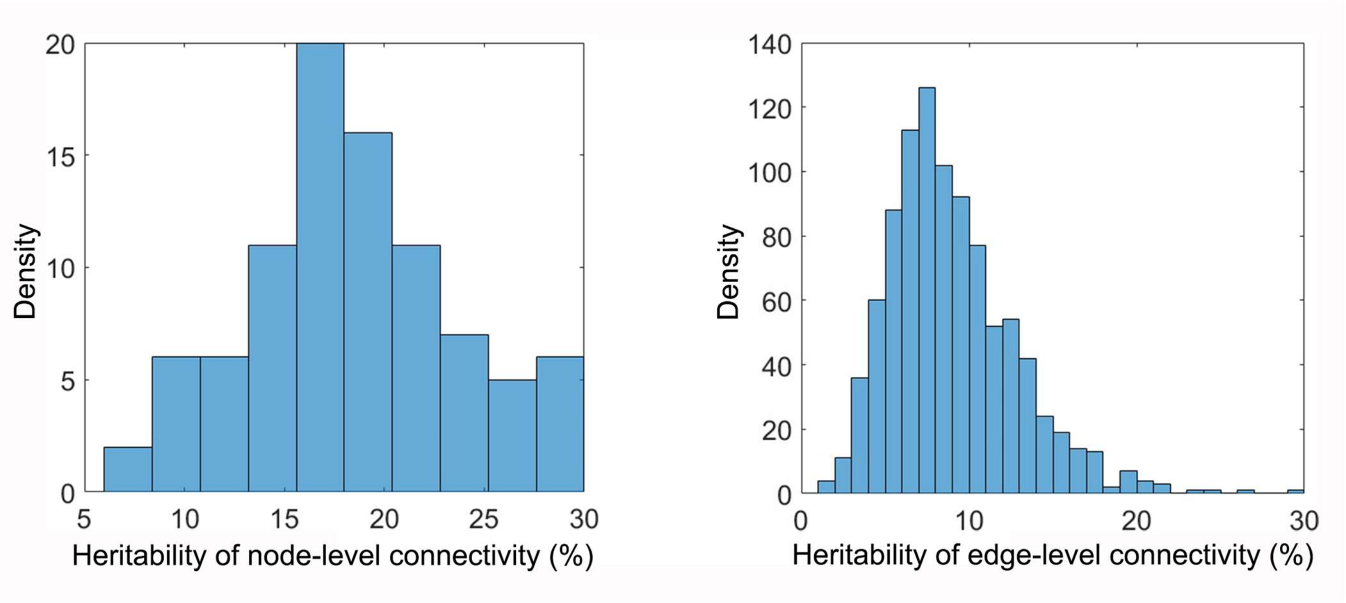

Supplementary Figure 2. Frequency histograms showing the distributions of SNP-based heritabilities, separately for 90 node-level connectivities (Left panel) and 947 edge-level connectivities (Right panel).

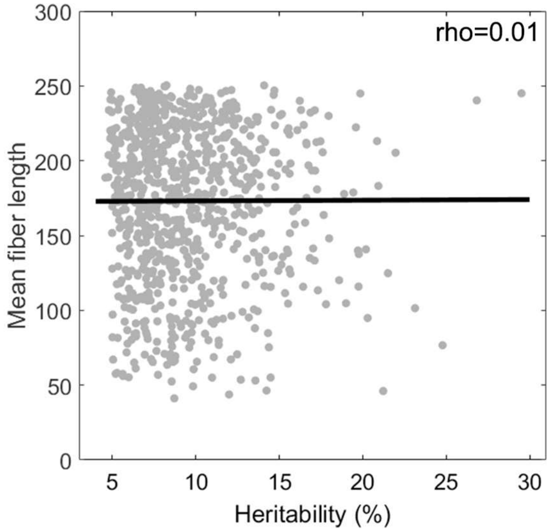

Supplementary Figure 3. No significant correlation between SNP-based heritabilities and fiber lengths of edge-level connectivities. Each dot represents an edge linking two brain regions defined in the AAL atlas.

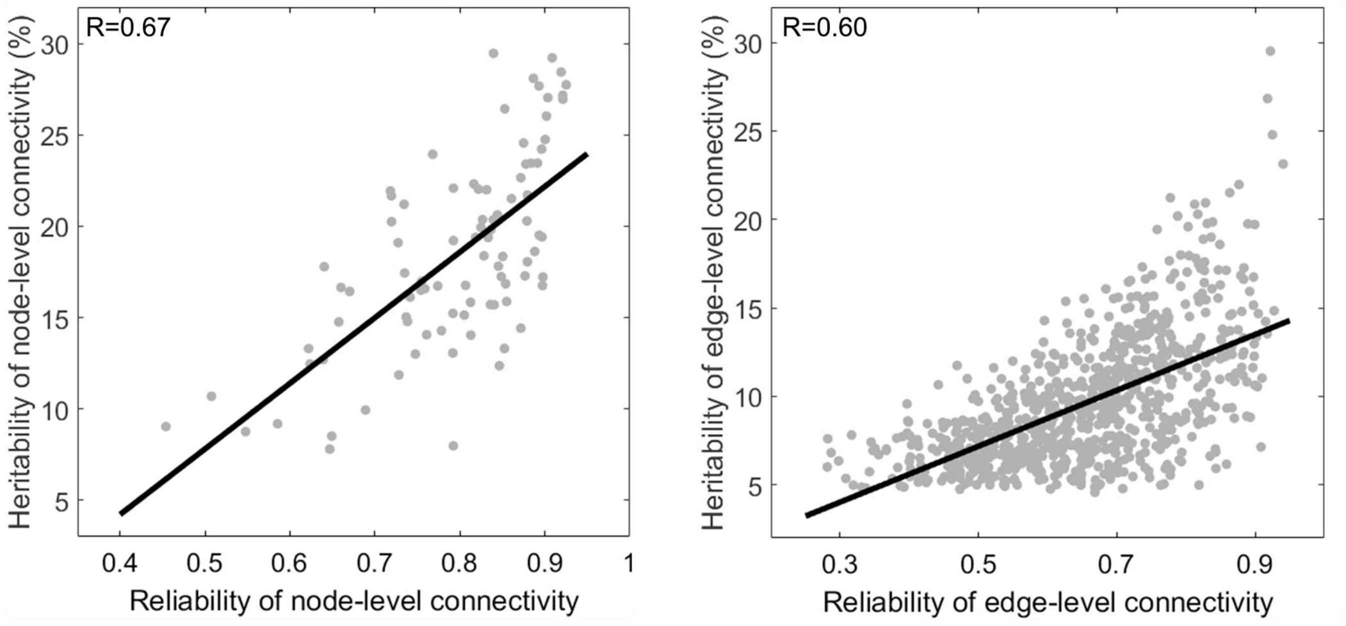

Supplementary Figure 4. Correlations between reliability (intra-class correlation) and heritability, separately for 90 node-level connectivities (left) and 947 edge-level connectivities (right).

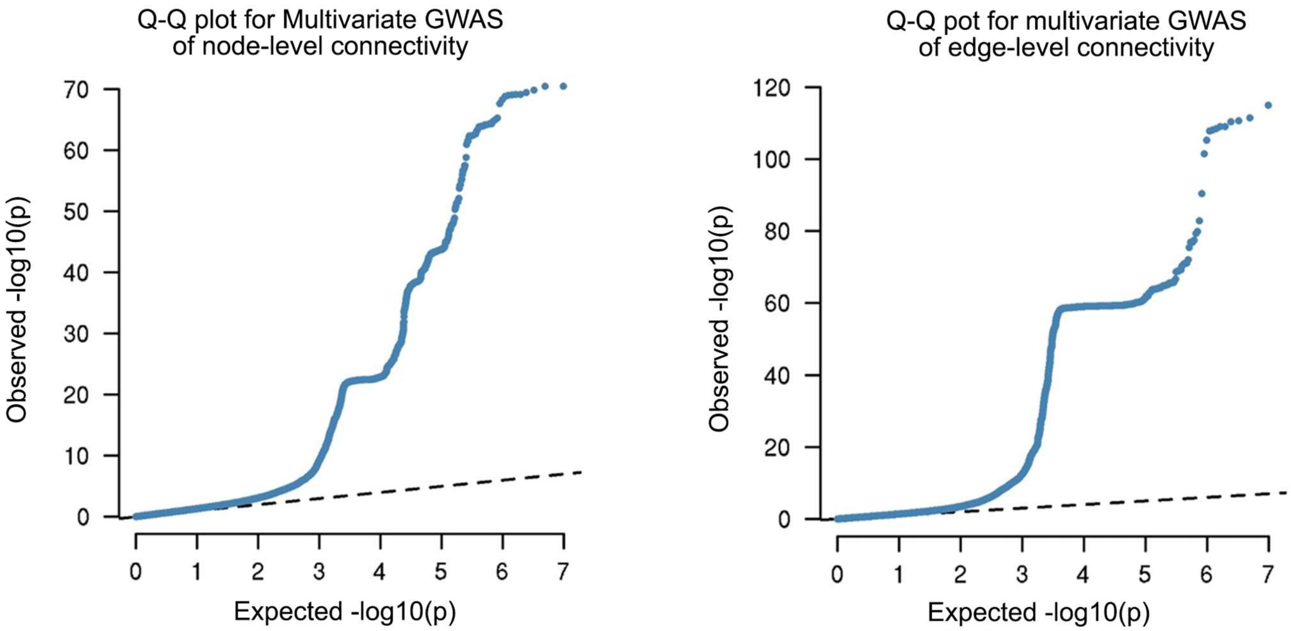

Supplementary Figure 5. Q-Q plots of multivariate GWAS for node-level connectivity and edge-level connectivity

Left panel: Q-Q plot of multivariate GWAS for node-level connectivity. Right panel: Q-Q plot of multivariate GWAS for edge-level connectivity. The black dotted line presents the observed –log10(p-values) from the multivariate GWAS plotted against the expected –log10(p-values) from a null distribution.

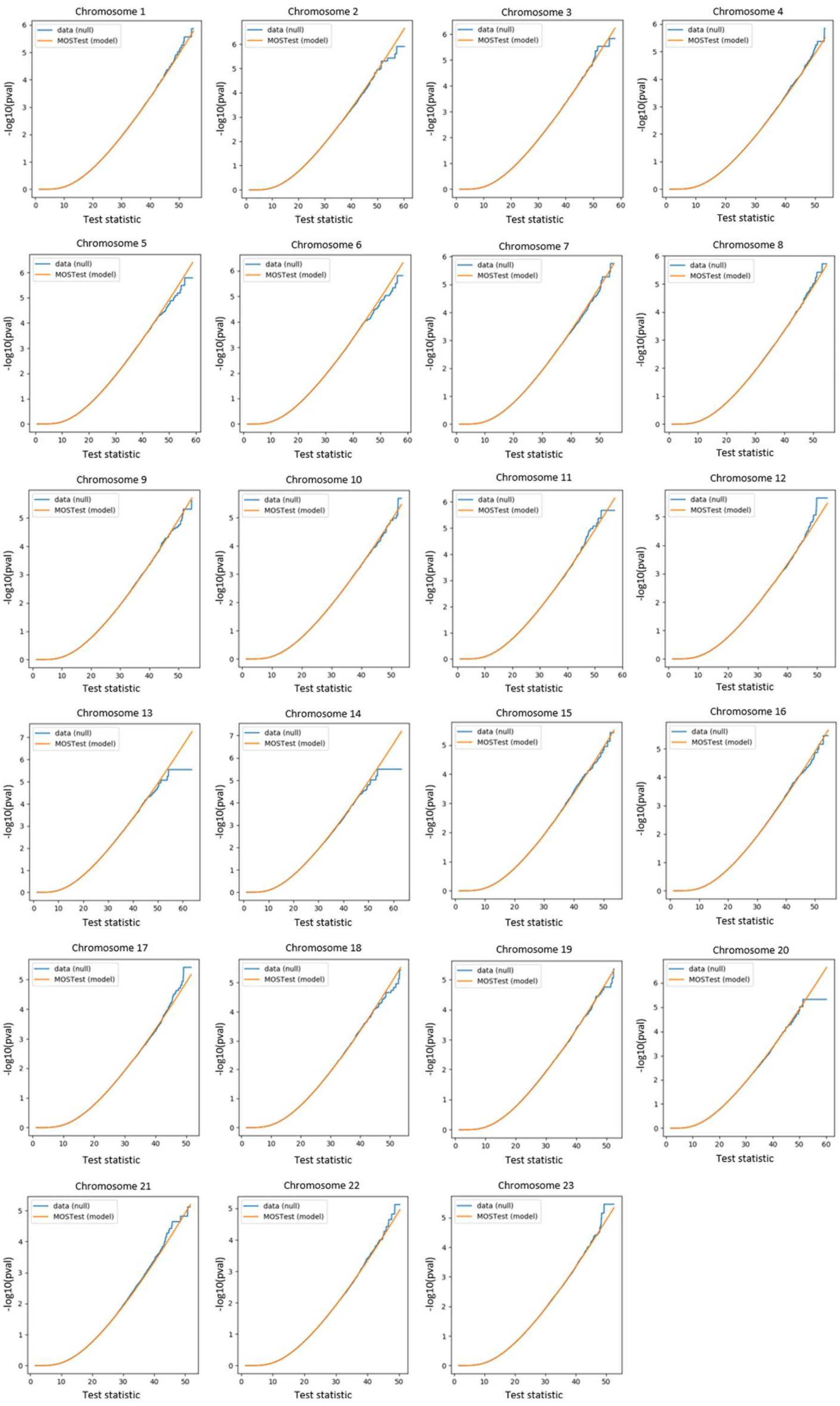

Supplementary Figure 6. Permuted and analytic P value distributions under the null, for the multivariate GWAS of node-level connectivity. Only null distributions are shown here, i.e. not the true association results. Close matching of the null p-value distributions from the permuted data (blue) and analytic forms (orange) indicate correct control of type 1 error. The analytic distribution can then be extrapolated to assess the significance of multivariate associations with far lower p values (see Methods). ‘Chromosome 23’ refers to chromosome X.

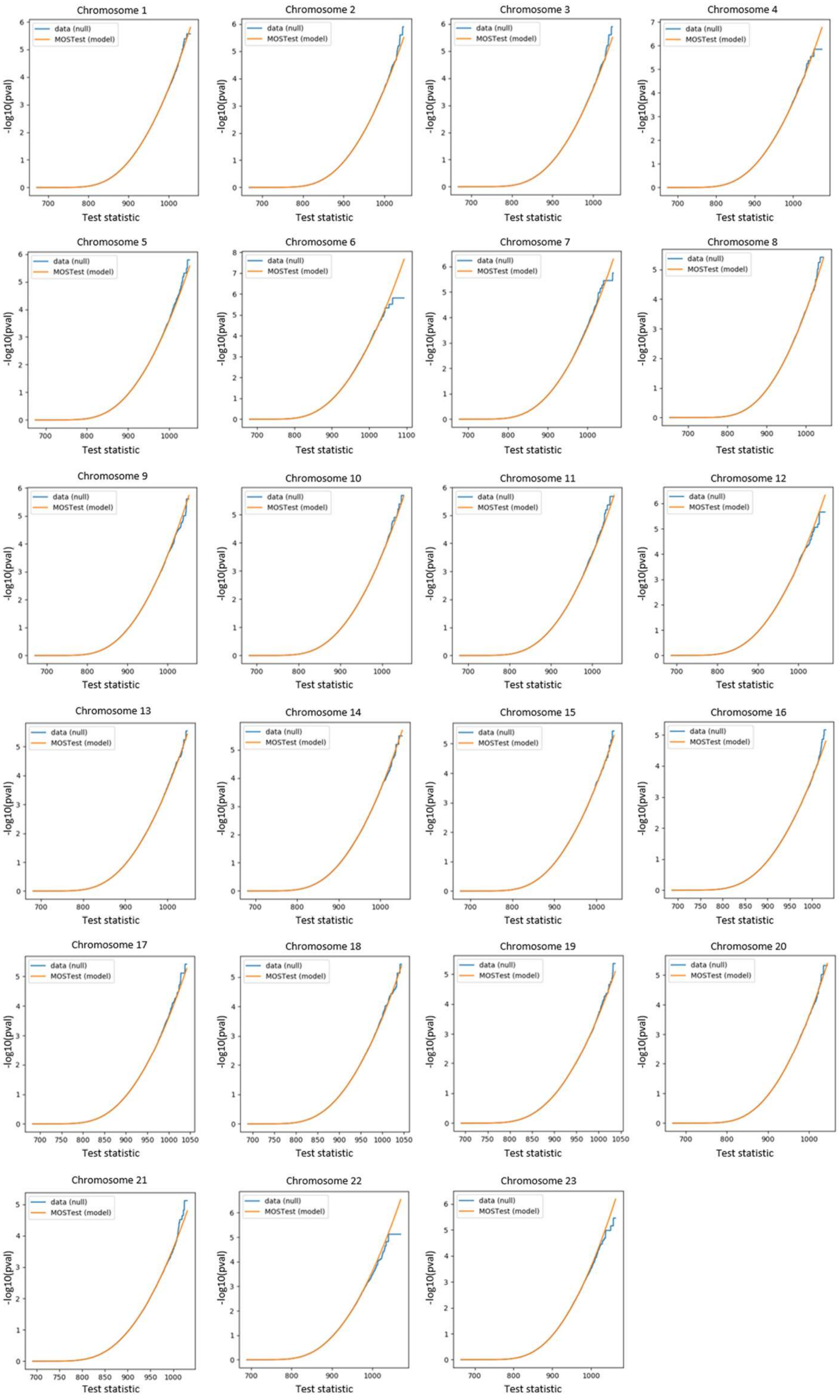

Supplementary Figure 7. Permuted and analytic P value distributions under the null, for the multivariate GWAS of edge-level connectivity. Only null distributions are shown here, i.e. not the true association results. Close matching of the null p-value distributions from the permuted data (blue) and analytic forms (orange) indicate correct control of type 1 error. The analytic distribution can then be extrapolated to assess the significance of multivariate associations with far lower p values (see Methods). ‘Chromosome 23’ refers to chromosome X.

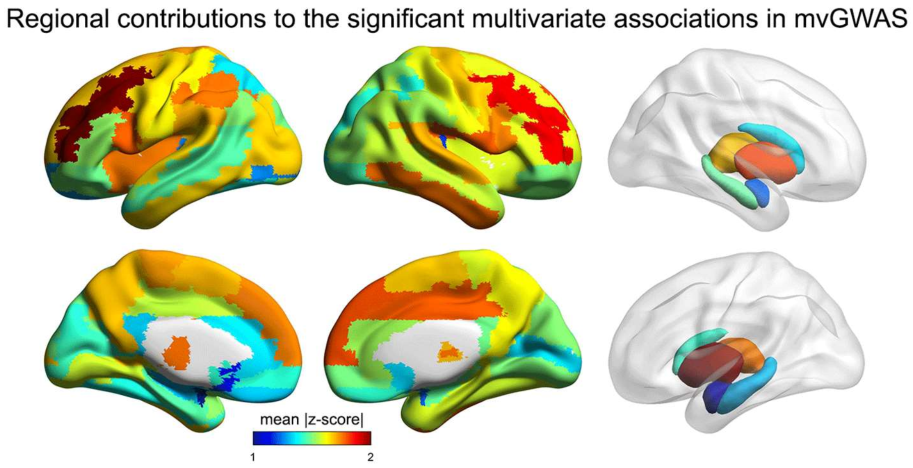

Supplementary Figure 8. Regional contributions to the significant multivariate associations in the node-level connectivity mvGWAS. For each region we calculated its average multivariate association z-score across all independently associated lead SNPs in the genome. This gives an indication of which node-level connectivities tend to be more often associated with genomic loci in general.

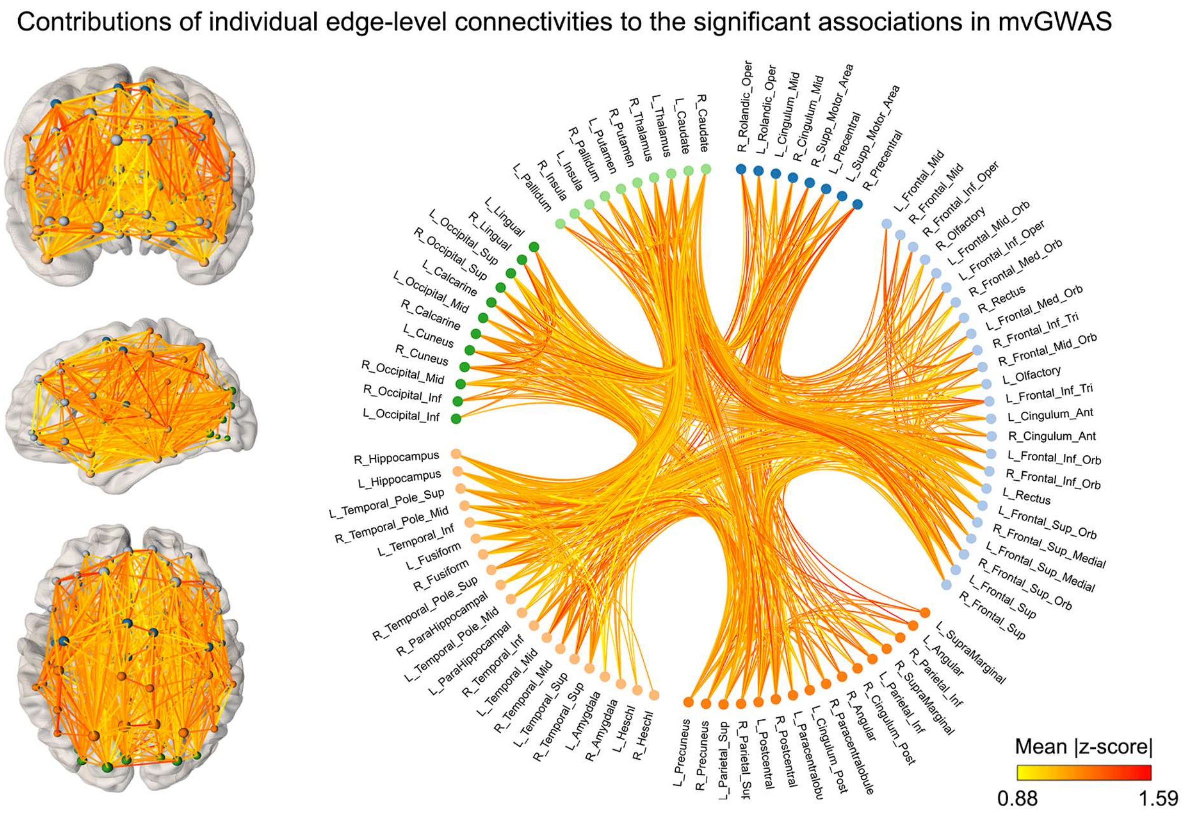

Supplementary Figure 9. Contributions of individual edge-level connectivities to the significant multivariate associations in mvGWAS. For each edge we calculated its average multivariate association z-score across all independently associated lead SNPs in the genome. This gives an indication of which edges tend to be more often associated with genomic loci in general. Left panel: brain maps. Right panel: nodes grouped by different anatomical lobes (frontal, prefrontal, parietal, temporal, occipital cortices, and subcortical structures).

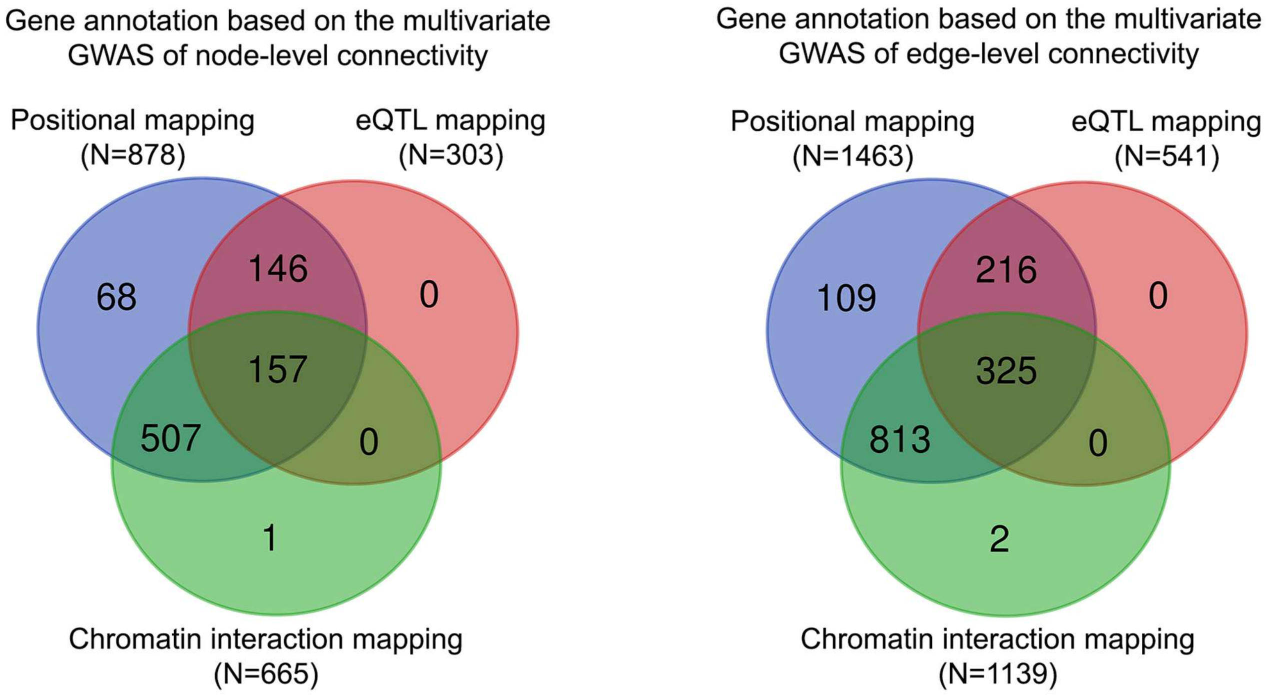

Supplementary Figure 10. Numbers of annotated genes at significantly associated genomic loci using three mapping strategies in FUMA. Left panel: annotated genes for significantly associated genomic loci in the multivariate GWAS of node-level connectivity. Right panel: annotated genes for significantly associated genomic loci in the multivariate GWAS of edge-level connectivity.

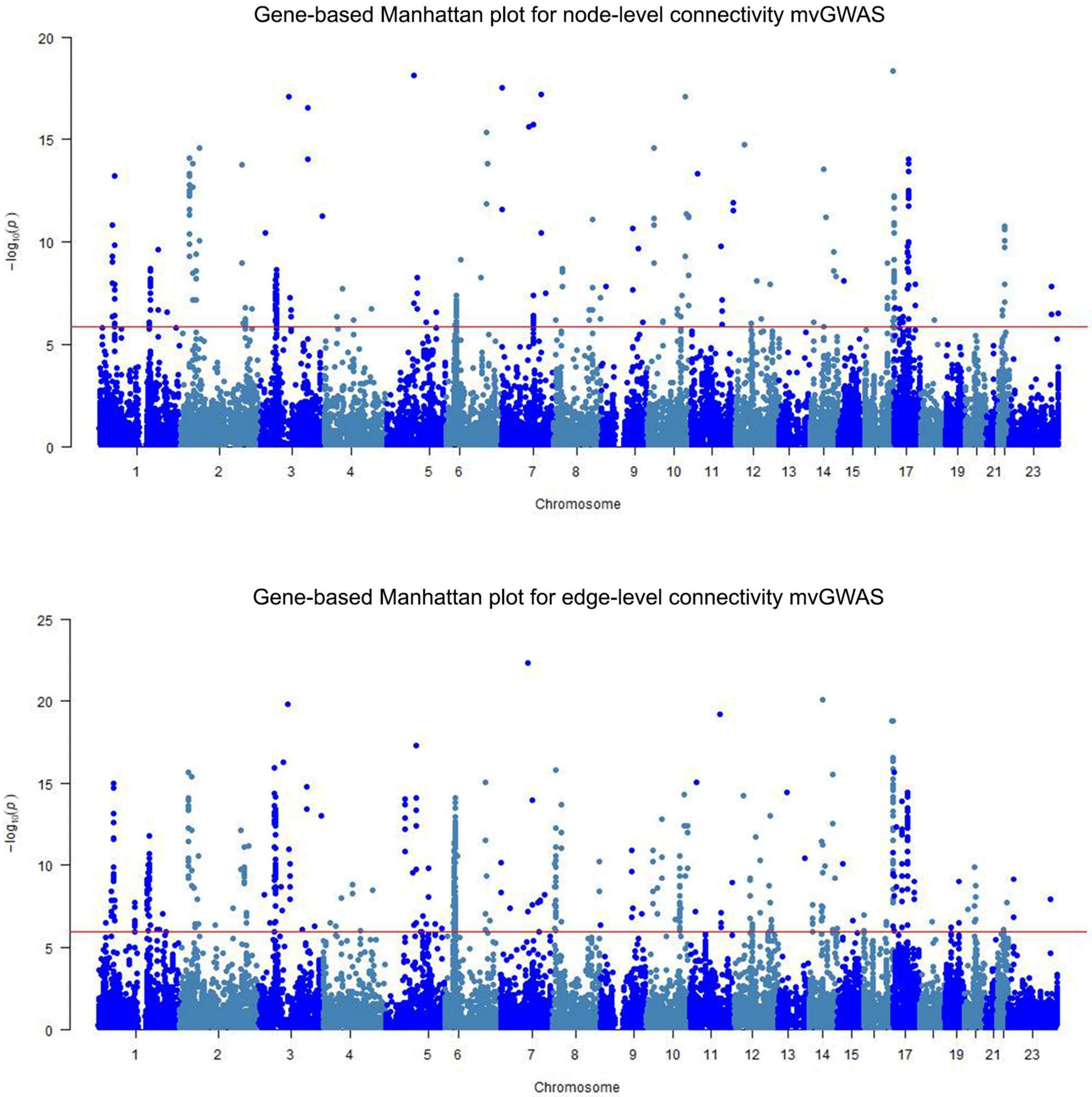

Supplementary Figure 11. Manhattan plots for genome-wide gene-based association analysis, separately using the node-level connectivity mvGWAS (upper) and edge-level connectivity mvGWAS (lower) results as input. The red line indicates the Bonferroni-corrected significance threshold for gene-based analysis of 20,146 genes (p<0.025/20,146).

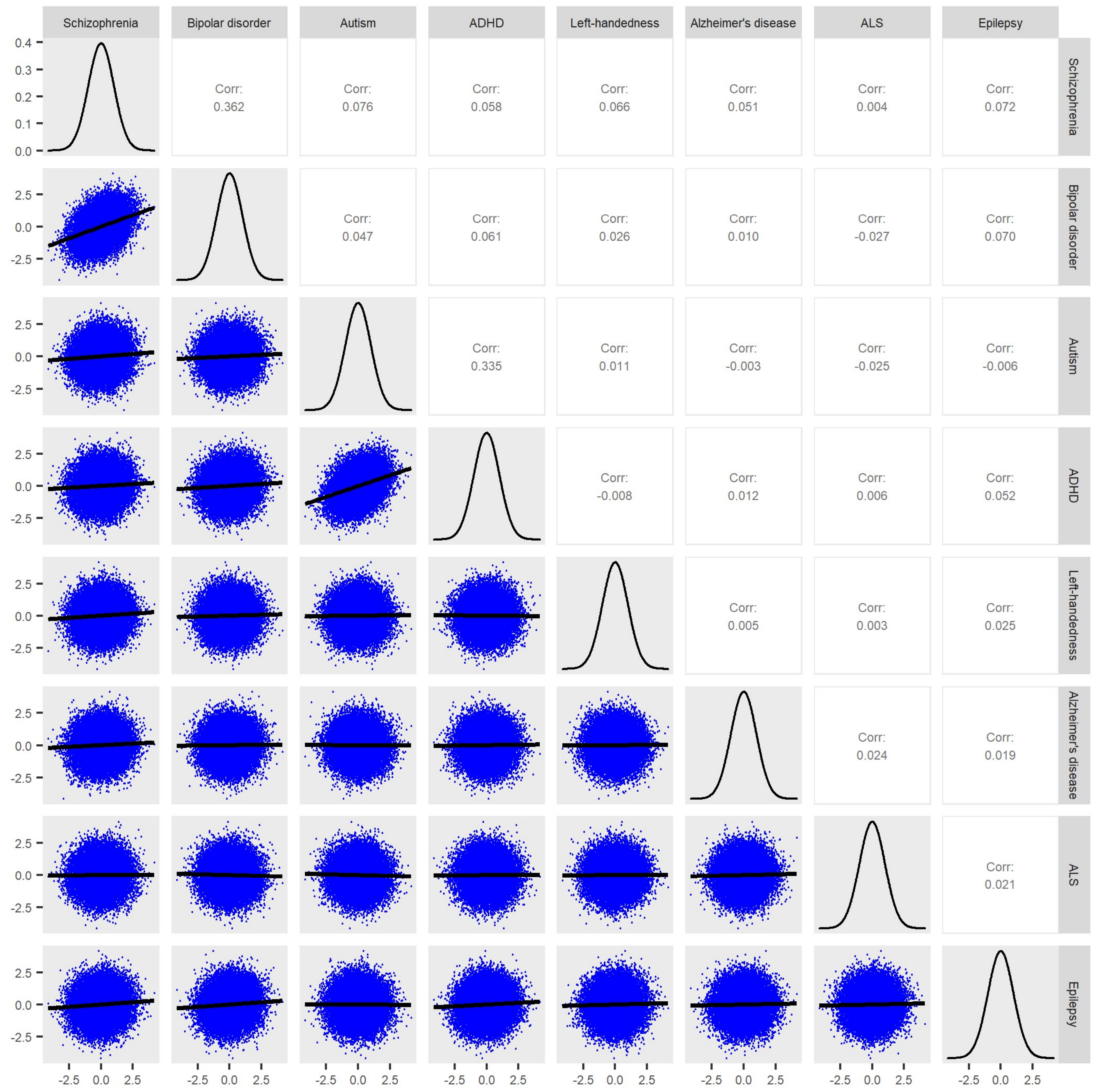

Supplementary Figure 12. Partial correlations between normalized polygenic scores for brain-related disorders or behavioral traits. Confounds including sex and age have been removed – see Methods. The diagonal of the matrix shows the distributions of the polygenic scores across the 30,810 individuals.

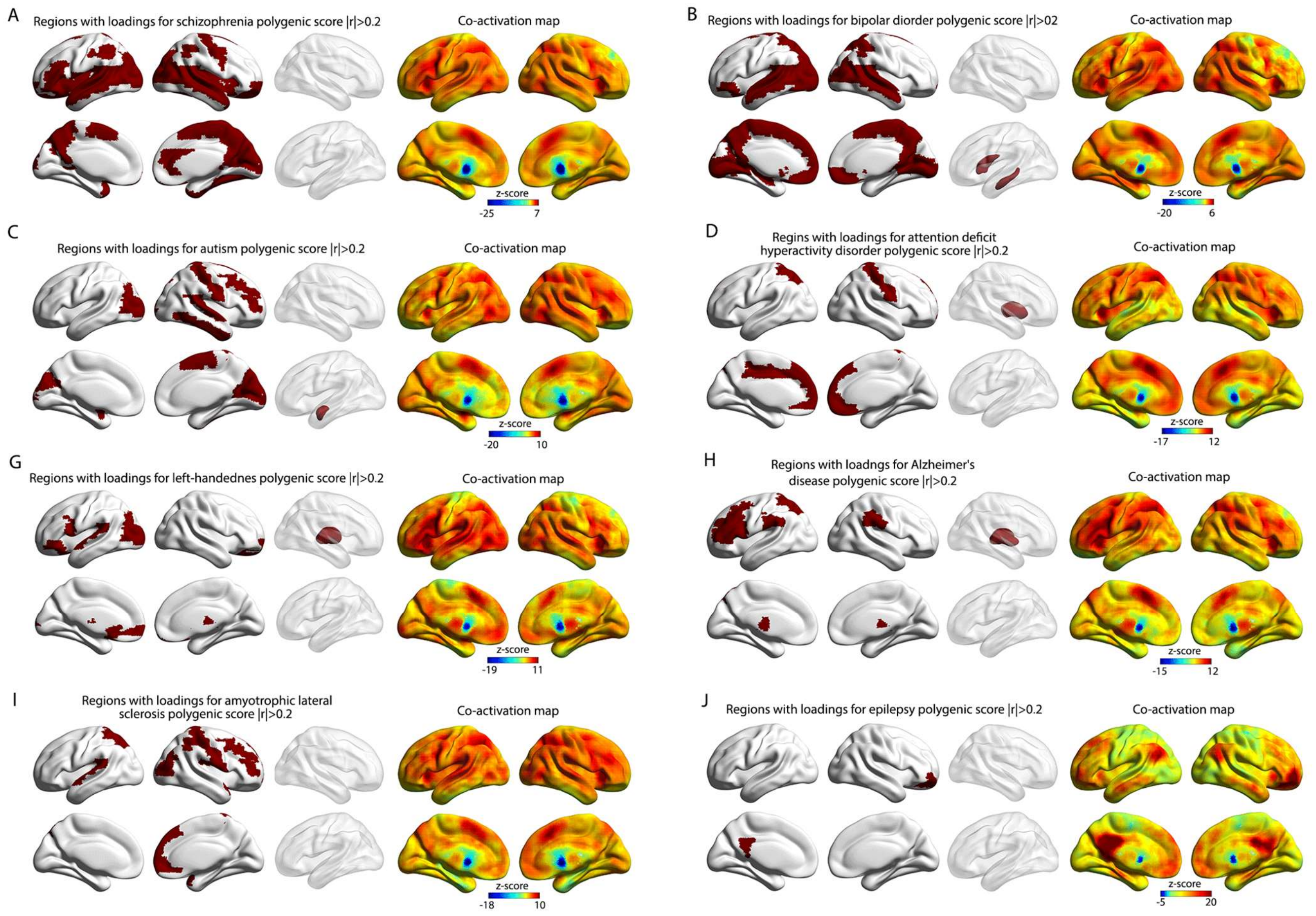

Supplementary Figure 13. Node-level connectivities showing the strongest associations with polygenic scores for brain-related disorders or behavioral traits. Left panel: Regions with loadings |r|>0.2 in canonical correlation analysis of their connectivities with a given polygenic score. The regional maps were used to define binary masks to query the Neurosynth database of meta-analyzed functional neuroimaging data from thousands of published studies. Right panel: Brain co-activation maps derived from the "decoder" function of Neurosynth, corresponding to the input masks.

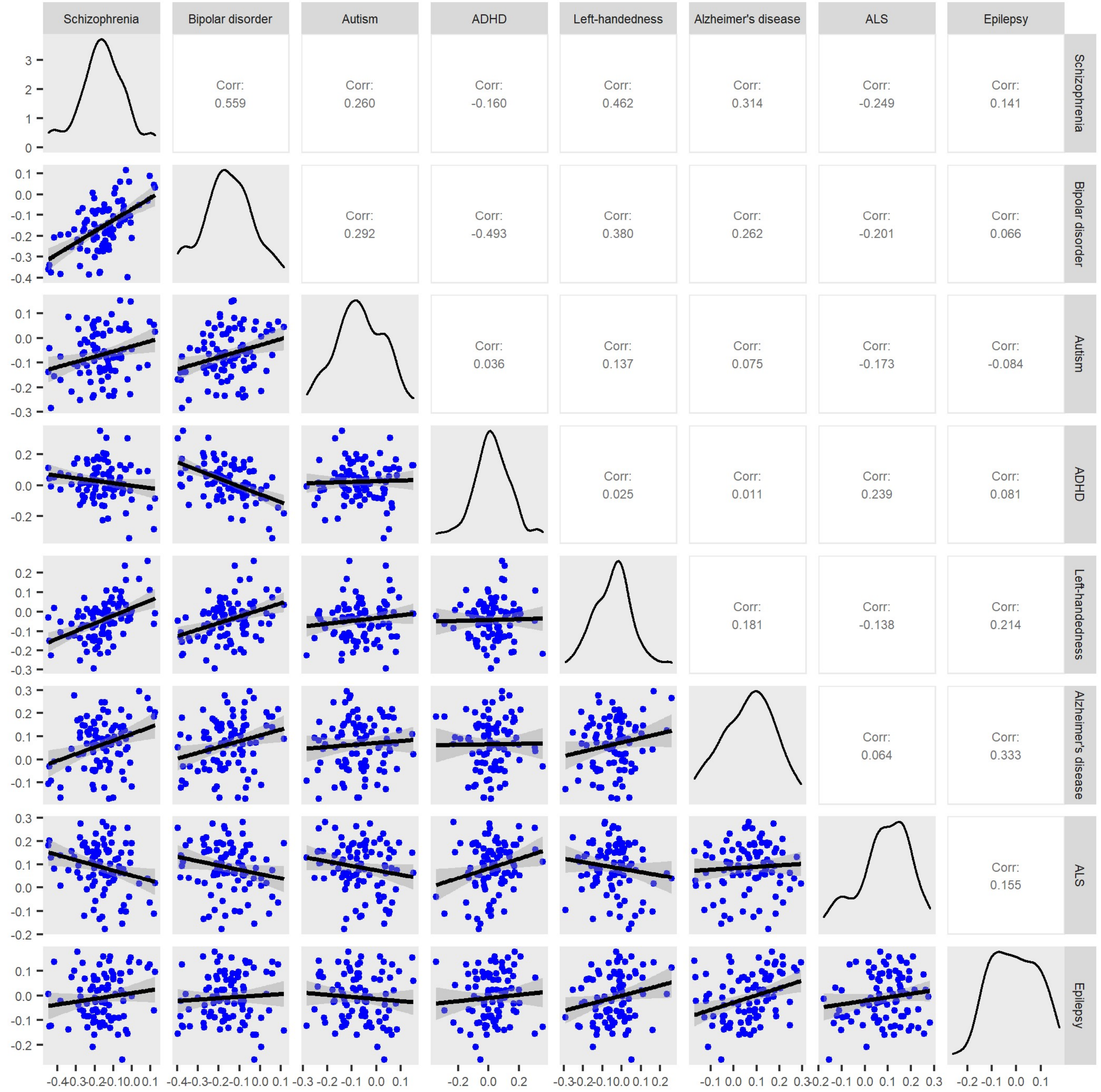

Supplementary Figure 14. Correlations between the loadings derived from different canonical correlation analyses between polygenic scores and node-level connectivities. These correlations are calculated over 90 brain regions. They show the extents to which polygenic dispositions to two different disorders/behaviors tend to associate consistently with the same brain regions. The diagonal of the matrix shows the distributions of loadings across regions.

Supplementary Tables 1-31. All supplementary tables are given in one separate Excel file. Their contents are as follows:
Supplementary Table 1. Node-level connectivity of the structural connectome.
Supplementary Table 2. SNP-based heritability estimates for node-level connectivity.
Supplementary Table 3. SNP-based heritability estimates for edge-level connectivity.
Supplementary Table 4. Significance of SNP-based heritability estimates for edge-level connectivities.
Supplementary Table 5. Standard errors of SNP-based heritability estimates for edge-level heritabilities.
Supplementary Table 6. 95% confidence interals of SNP-based heritability estimates for edge-level heritabilities.
Supplementary Table 7. Intraclass correlation coefficients from reliability analysis for node-level connectivity.
Supplementary Table 8. Intraclass correlation coefficients from reliability analysis for edge-level connectivity.
Supplementary Table 9. Lead SNPs from multivariate GWAS of node-level connectivity.
Supplementary Table 10. Lead SNPs from multivariate GWAS of edge-level connectivity.
Supplementary Table 11. Univariate association z scores for each lead SNP and node-level connectivities.
Supplementary Table 12. Univariate association z scores for each lead SNP and edge-level connectivities.
Supplementary Table 13. Regional contributions to the significant multivariate associations from node-level connectivity mvGWAS.
Supplementary Table 14. The contributions of individual edges to the significant multivariate associations from edge-level connectivity mvGWAS.
Supplementary Table 15. Lead SNPs associated with either node-level connectivity or edge-level connectivity in the present study that were also associated with white matter microstructure in reference *10* (see main text).
Supplementary Table 16. Gene mapping at all significant node-level connectivity mvGWAS loci based on three strategies.
Supplementary Table 17. Gene mapping at all significant edge-level connectivity mvGWAS loci based on three strategies.
Supplementary Table 18. Significant genes from gene-based analysis of node-level connectivity mvGWAS results.
Supplementary Table 19. Results from MAGMA gene set analysis, using node-level connectivity multivariate GWAS as input.
Supplementary Table 20. Significant genes from gene-based analysis of edge-level connectivity multivariate GWAS results.
Supplementary Table 21. Results from MAGMA gene set analysis, using edge-level connectivity multivariate GWAS as input.
Supplementary Table 22. Differential expression of genes associated with node-level connectivity in different lifespan stages from BrainSpan brain samples.
Supplementary Table 23. Differential expression of genes associated with edge-level connectivity in different lifespan stages from BrainSpan brain samples.
Supplementary Table 24. Differential expression of genes associated with node-level connectivity in different cell types in the developing human brain.
Supplementary Table 25. Differential expression of genes associated with edge-level connectivity in different cell types in the developing human brain.
Supplementary Table 26. Lead SNPs showing significant association with at least one core language network tract connectivity, and genes annotated to these lead SNPs by genomic position, eQTL and chromatin interaction.
Supplementary Table 27. Correlations between polygenic scores for brain-related disorder or behavioural traits.
Supplementary Table 28. Loadings from the canonical correlation analyses between node-level connectivities and polygenic dispositions to brain-related disorders or behavioural traits.
Supplementary Table 29. Loadings from the canonical correlation analyses between node-level connectivities and polygenic dispositions for Alzheimer's disease after excluding the APOE locus.
Supplementary Table 30. Functional terms based on meta-analyzed fMRI data, associated with co-activation maps for brain regions where node-level connectivity had loadings $|r|>0.2$ for polygenic scores of brain-related disorders or behavioural traits.
Supplementary Table 31. Correlation coefficients (across 90 regions) between loadings from multivariate associations of node-level connectivities with polygenic dispositions to brain-related disorders or behavioural traits.
